# DNA methylation of chronic lymphocytic leukemia with differential response to chemotherapy

**DOI:** 10.1038/s41597-020-0456-0

**Published:** 2020-05-01

**Authors:** Deyan Yordanov Yosifov, Johannes Bloehdorn, Hartmut Döhner, Peter Lichter, Stephan Stilgenbauer, Daniel Mertens

**Affiliations:** 1 Department of Internal Medicine III, Ulm University, Ulm, Germany; 2 Cooperation Unit “Mechanisms of Leukemogenesis”, German Cancer Research Center (DKFZ), Heidelberg, Germany; 3 Division of Molecular Genetics, German Cancer Research Center (DKFZ), Heidelberg, Germany

**Keywords:** Chronic lymphocytic leukaemia, DNA methylation

## Abstract

Acquired resistance to chemotherapy is an important clinical problem and can also occur without detectable cytogenetic aberrations or gene mutations. Chronic lymphocytic leukemia (CLL) is molecularly well characterized and has been elemental for establishing central paradigms in oncology. This prompted us to check whether specific epigenetic changes at the level of DNA methylation might underlie development of treatment resistance. We used Illumina Infinium HumanMethylation450 BeadChips to obtain DNA methylation profiles of 71 CLL patients with differential responses. Thirty-six patients were categorized as relapsed/refractory after treatment with fludarabine or bendamustine and 21 of them had genetic aberrations of *TP53*. The other 35 patients were untreated at the time of sampling and 15 of them had genetic aberration of *TP53*. Although we could not correlate chemoresistance with epigenetic changes, the patients were comprehensively characterized regarding relevant prognostic and molecular markers (e.g. IGHV mutation status, chromosome aberrations, *TP53* mutation status, clinical parameters), which makes our dataset a unique and valuable resource that can be used by researchers to test alternative hypotheses.

## Abstract (summary)

**Table:** 

Measurement(s)	DNA methylation
Technology Type(s)	methylation profiling by array
Factor Type(s)	TP53 mutation status • response to fludarabine or bendamustine • chromosomal aberration • IGHV mutation status
Sample Characteristic - Organism	Homo sapiens

Machine-accessible metadata file describing the reported data: 10.6084/m9.figshare.12006624

## Background & Summary

Chronic lymphocytic leukemia (CLL) is the most common leukemia in the Western world and mainly affects elderly patients^1^. Its incidence rate was 8.3 cases per 100 000 men and 5.8 cases per 100 000 women in Germany in 2014^2^. CLL is characterized by accumulation of small B lymphocytes with a mature appearance in blood, bone marrow, lymph nodes and other lymphoid tissues^3^. The clinical course of CLL differs depending on the biological characteristics of the disease (hypermutation status of the immunoglobulin heavy-chain genes (IGHV), presence of specific genomic aberrations and/or recurrent mutations in oncogenes and tumor suppressor genes)^4–6^. Some of these genetic features are associated with distinct epigenetic profiles, e.g. CLL tumours with high level of IGHV somatic hypermutation (M-CLL) have distinct DNA methylation patterns compared to CLL tumours with a low or absent IGHV mutational load (U-CLL)^7^.

Chemoimmunotherapeutic regimens like fludarabine, cyclophosphamide and rituximab (FCR) or bendamustine and rituximab (BR) achieve durable remissions in the majority of treatment-naïve CLL patients^8–11^. Although novel targeted and effective treatments for CLL were introduced in the past five years, FCR is not inferior to them as first-line therapy in the subgroup of young and fit patients with M-CLL without 17p deletion and/or *TP53* mutation (del(17p)/*TP53*mut)^12,13^. Additionally, the high cost of novel targeted drugs limits their use in developing countries where conventional cytotoxic chemotherapy is still a viable option^14,15^. Thus, drugs like fludarabine and bendamustine will continue to be used in the future for treatment of CLL and development of resistance to these classical chemotherapeutics remains an important problem to study.

**Fig. 1:**
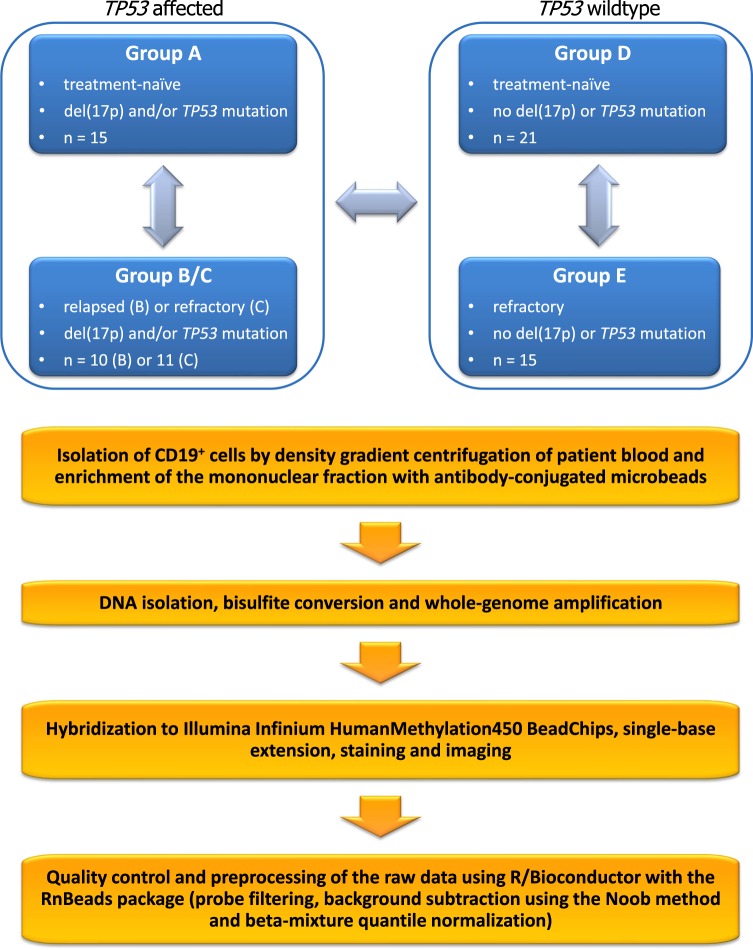
Schematic overview of the study design and experimental procedure.

Chemorefractoriness of CLL is most often caused by functional impairment of the ATM-p53 DNA damage response pathway, mostly as a result of cytogenetic aberrations or mutations^16,17^. Del(17p) is found in 5% to 10% of patients at diagnosis but in up to 40% of patients relapsing after fludarabine-based treatment regimens^18^. Del(17p) causes loss of one allele of the tumour suppressor *TP53* but in about 80% of the cases the other allele is also inactivated by somatic mutation^6,18^. Nevertheless, even monoallelic aberrations of *TP53* confer poor prognosis. Interestingly, some cases of chemorefractory CLL show dysfunction of the ATM-p53 pathway without respective genetic lesions^16,17^. Additional genes and pathways have been implicated in development of resistance to fludarabine, although also in these cases mutations are not always detectable^17,19,20^. These observations leave the possibility that chemoresistance in CLL can also be driven by epigenetic mechanisms. In order to find epigenetic changes associated with chemoresistance, we selected samples from patients that were relapsed/refractory after treatment with fludarabine or bendamustine and/or had del(17p)/*TP53*mut, as well as samples from CLL patients without del(17p)/*TP53*mut who had treatment-naïve disease or who achieved prolonged remission after treatment with fludarabine- or bendamustine-based regimens. The grouping of the samples is shown in Fig. 1. This selection of samples allows comparing relapsed/refractory patients to untreated patients after stratification for the presence or absence of aberrations affecting the *TP53* locus. In our opinion, this stratification is important because presence of *TP53* aberrations could obscure the effect of epimutations, as *TP53* aberrations themselves are a strong determinant of chemoresistance^8,21,22^. On the other hand, the chosen design of the study could allow to detect epimutations that additionally occur in the subgroup of *TP53*-disrupted CLL tumours to further reduce their sensitivity to chemotherapy. Genome-wide DNA methylation in all selected samples (N = 72) was quantified using Illumina Infinium HumanMethylation450 BeadChips. The resulting raw signal data and a normalized data matrix are provided here as a resource for studying relationships between epigenetics and chemoresistance in CLL. Basic clustering and principal component analyses did not intuitively show grouping of samples according to chemoresistance status. However, we cannot exclude that more sophisticated analyses will be able to extract relevant differences and correlations. Notably, the dataset is unique with the high proportion of patients with del17p and/or mutated *TP53*. This dataset thus allows comparison of epigenetic profiles of CLL patients with negative prognostic markers to profiles of patients with chemosensitive CLL and CLL not harbouring *TP53* defects.

## Methods

### Patient sample selection and molecular characterization

The biological and molecular characteristics of the 71 CLL patients included in the study are listed in Table 1 and Online-only Table 1. Fifty-one of the patients were subjects of the multi-centre CLL2O clinical trial (clinicaltrials.gov: NCT01392079) and were subdivided here into 4 subgroups depending on their del(17p)/*TP53*mut and treatment/response statuses as follows: groups A (N = 15), B (N = 10) and C (N = 11) consisted of patients with del(17p) and/or *TP53* mutation and group E (N = 15) consisted of patients without del(17p) or *TP53* mutation. Patients in group A were not treated previously but required treatment, patients in group B had relapsed after treatment with fludarabine- or bendamustine-containing regimens and patients in groups C and E were refractory to fludarabine or bendamustine. Additional 20 cases (group D) were patients whose tumours did not harbour del(17p) or *TP53* mutation and who were not previously treated but some of whom required treatment and responded to subsequent therapy with fludarabine- or bendamustine-containing regimens (N = 6, Online-only Table 1). All patients had a confirmed diagnosis of CLL by flow cytometry; their IGHV mutational status and cytogenetics were also determined during the diagnostic workup. Unmutated IGHV gene (≥98% homology to germline) was detected by sequencing in 58 patients (81.7%). Fluorescent *in-situ* hybridization (FISH) analysis revealed the presence of del(17p) in 35 of a total 36 patients in groups A, B and C, as well as the absence of such an aberration in all patients from groups D and E. Mutated *TP53* was detected in 30 of total 36 patients in groups A, B and C, and in none of the patients in groups D and E. One of the patients in group D had two consecutive samples taken with a time difference of 40 months (Online-only Table 1). All patients provided informed consent to subsequent analysis and research in accordance with the Declaration of Helsinki and under a protocol approved by the ethical committee of the University of Ulm.

**Table 1:** Biological and molecular features of CLL patients included in the study.

Characteristic	All patients (N = 71)	A (del(17p), untreated, N = 15)	B (del(17p), relapsed, N = 10)	C (del(17p), refractory, N = 11)	D (no del(17p), chemosensitive, N = 20)	E (no del(17p), refractory, N = 15)	P Value*
Age at sampling, years							0.27
Median	64	66	62	67	62	63	
Range	38–84	49–77	53–68	54–76	38–84	38–71	
IGHV mutational status, number (%)							0.45
Mutated (<98% homology)	13 (18.3)	2 (13.3)	0 (0)	3 (27.3)	5 (25)	3 (20)	
Unmutated (≥98% homology)	58 (81.7)	13 (86.7)	10 (100)	8 (72.7)	15 (75)	12 (80)	
FISH analysis, number (%)							P < 0.0001
Normal karyotype	12 (16.9)	0 (0)	0 (0)	0 (0)	10 (50)	2 (13.3)	
Del(13q14)	46 (64.8)	9 (60)	6 (60)	8 (72.7)	10 (50)	13 (86.7)	
Trisomy 12	7 (9.9)	3 (20)	1 (10)	3 (27.3)	0 (0)	0 (0)	
Del(11q22)	13 (18.3)	3 (20)	3 (33.3)	3 (27.3)	0 (0)	4 (26.7)	
Del(17p13)	35 (49.3)	15 (100)	10 (100)	10 (90.9)	0 (0)	0 (0)	
*TP53* mutational status, number (%)							P < 0.0001
Mutated	30 (42.3%)	10 (66.7)	9 (90)	11 (100)	0 (0)	0 (0)	
Unmutated	41 (57.7%)	5 (33.3)	1 (10)	0 (0)	20 (100)	15 (100)	

*All P values are for comparisons across all five groups and are two-sided. P values for numerical variables were calculated with the use of the Kruskal–Wallis test, and P values for categorical variables were calculated with the use of the chi-square test or Fisher’s exact test. FISH profiles were summarized according to the hierarchical risk model^4^ before performing the test.

### Sample preparation

Blood samples from CLL patients were subjected to density gradient centrifugation (Pancoll human, #P04-60500, PAN-Biotech, Germany) to isolate peripheral blood mononuclear cells (PBMCs), which were then enriched for CD19+ B cells using CD19 MicroBeads (#130-050-301, Miltenyi Biotec, Germany) and LS columns (#130-042-401, Miltenyi Biotec). The purity of the enriched cell fractions was confirmed using a FACSCalibur flow cytometer (Becton Dickinson & Co.) and a monoclonal mouse anti-human CD19 antibody (clone HD37, DakoCytomation, Denmark). Purified cell samples were flash frozen and stored as dry cell pellets at −80 °C for further analysis.

### DNA extraction, bisulfite conversion and methylation level quantification

The 72 frozen cell pellets were processed in 6 batches, taking care that samples from each of the 5 subgroups (A-E) were approximately equally divided among the 6 batches to mitigate possible batch effects. DNA was extracted from the cell pellets by the Qiagen AllPrep kit (#80204) and quantification and quality control were performed using a NanoDrop ND-1000 UV-Vis Spectrophotometer (Thermo Scientific, USA). One and a half micrograms of DNA from each sample were sent to the Genomics and Proteomics Core Facility of the German Cancer Research Center (DKFZ) for bisulfite conversion and hybridization to Illumina Infinium HumanMethylation450 BeadChips, according to the manufacturer’s instructions. The bisulfite conversion was performed using the EZ DNA Methylation Kit (Zymo Research) and then the converted DNA was whole-genome amplified and fragmented. The processed samples were distributed randomly among 6 Illumina Infinium HumanMethylation450 BeadChips. The core facility was blinded regarding the identity of the samples and the experimental groups to which they belonged. After hybridization, single-base extension and staining, BeadChips were scanned using an Illumina iScan reader, and the fluorescence intensity raw data for each sample was recorded as two IDAT files, one for the green (Cy3) and one for the red (Cy5) channel^23^. Quality control of the whole procedure was performed using the Methylation Module of Illumina’s GenomeStudio software.

### Data processing and statistics

After acquiring the raw data, we performed quality control, preprocessing and basic analysis using R/Bioconductor with the RnBeads package^24^. Illumina probes known to be cross-reactive or overlapping known SNPs^25^ were excluded from analysis. This was also done for probes giving unreliable measurements as determined by the Greedycut algorithm implemented in RnBeads. The data from the remaining probes were subjected to background subtraction using the Noob method^26^ and beta-mixture quantile normalization (BMIQ)^27^. In a subsequent step, probes of non-CpG context, probes binding to sequences on sex chromosomes and probes with low standard deviation were filtered out. CpG sites on the sex chromosomes were excluded to avoid gender-specific methylation bias, as groups within our study did not contain equal numbers of males and females. CpG sites with low standard deviation are generally not informative and removing them from the analysis is a common approach to increase power for detection of differentially methylated CpGs and to improve sensitivity of clustering^28,29^. The data obtained by the remaining probes^23^ were used in downstream analyses. Methylation levels of CpG sites were calculated as β-values (β = intensity of the methylated allele (M)/[intensity of the unmethylated allele (U) + intensity of the methylated allele (M) + 100].

Both multidimensional scaling (MDS) and principal-component analysis (PCA) were used as dimension reduction techniques. Hierarchical clustering was carried out using the Manhattan distance metric and complete linkage criteria.

## Data records

The complete DNA methylation microarray dataset has been deposited in the NCBI Gene Expression Omnibus (GEO) database and consists of the raw data in the form of 72 pairs (red/green fluorescence) of raw Intensity Data files (.idat), the processed data matrix and a metadata table describing the samples and their groups^23^. For convenience, Online-only Table 1 lists all patients and samples with their characteristics, as well as experimental and analytical procedures and output data file names.

## Technical Validation

### Quality control of genomic DNA

Genomic DNA 260 nm/280 nm absorbance ratios were determined using a NanoDrop ND-1000 UV-Vis Spectrophotometer (Thermo Scientific, USA). All samples had ratios in the range 1.8–2.0, as expected for DNA of high purity (Online-only Table 2).

### Quality control of bisulfite conversion and Infinium 450k data

**Fig. 2:**
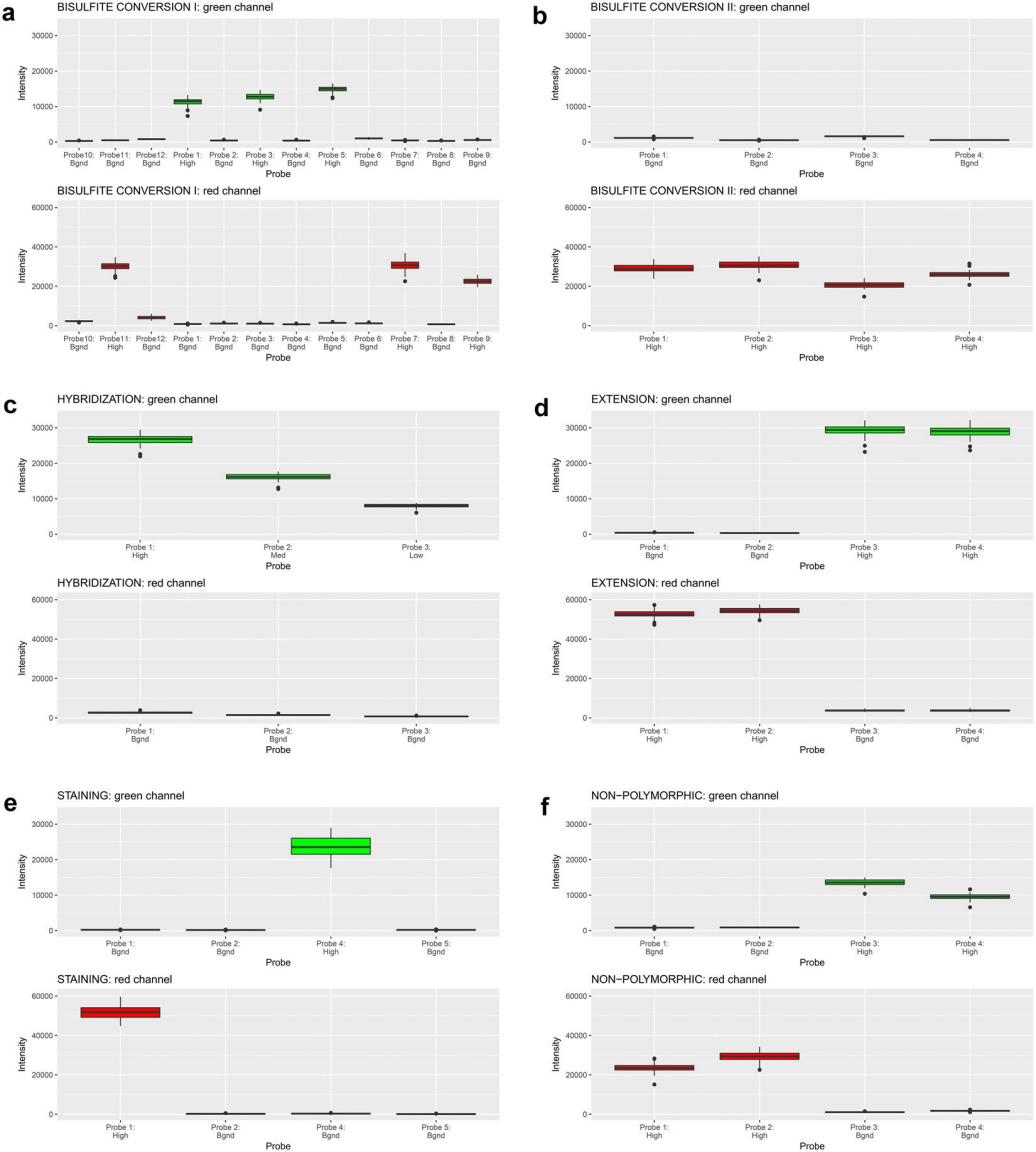
Distribution (median and range) of signal intensity for quality control probes on Illumina Infinium HumanMethylation450 arrays across all samples and in each of the colour channels (green/red). **(a,b)** Bisulfite conversion efficiency as reported by control probes of Infinium design I **(a)** or II **(b)**. **(c)** Hybridization performance using synthetic reference targets present in the hybridization buffer at three concentrations. **(d)** Efficiency of extension of A, T, C and G nucleotides from hairpin probes (sample-independent). Probe 1 is specific for A, probe 2 for T, probe 3 for C and probe 4 for G. **(e)** Efficiency and sensitivity of the staining step (independent of the hybridization and extension steps). **(f)** Overall efficiency of the procedure estimated by querying non-polymorphic bases in the genome – one probe for each nucleotide. In all plots, labels give the expected intensity level: high, medium (Med), low or background (Bgnd).

Quality control of bisulfite conversion and of data obtained by the Illumina Infinium HumanMethylation450 BeadChips was performed independently by the team of the core facility using the Methylation Module of Illumina’s GenomeStudio software (Supplementary File 1 and Online-only Table 3) and by us using the rnb.run.qc command of the RnBeads package (Fig. 2). Both analyses ascertained the correct execution of the separate steps of the whole experimental procedure: bisulfite conversion, hybridization, single-base extension and stripping. Figure 2a,b demonstrate bisulfite conversion efficiency as reported by control probes of Infinium I or II design, respectively. Overall hybridization performance was assessed using synthetic reference targets that are present in the hybridization buffer at three concentrations (low, medium and high) and that resulted in signals with well separable intensity intervals, as expected (Fig. 2c). The extension controls showed high efficiency of extension with any of the 4 nucleotides (Fig. 2d) and the staining controls demonstrated high efficiency and sensitivity of the staining step (Fig. 2e). The overall performance of the assay from amplification to detection is summarized by the signal from probes that query non-polymorphic bases in the genome – one probe for each nucleotide (Fig. 2f).

**Fig. 3:**
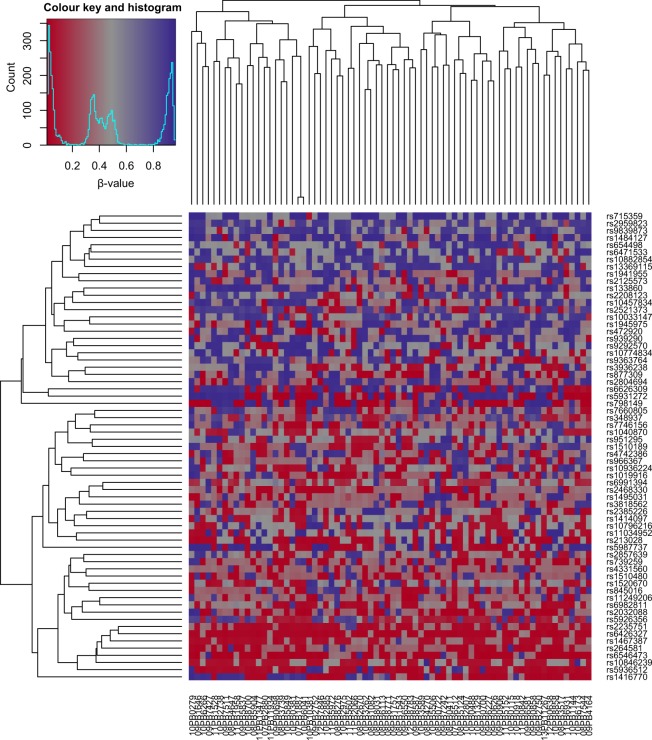
Heatmap of signal from the 65 probes on the methylation arrays that distinguish single nucleotide polymorphisms (SNPs). Two samples show highly similar patterns, as they originate from the same patient.

The Infinium 450k BeadChip contains 65 genotyping probes that are useful for identification of sample mix-ups. These probes produced highly similar signal patterns in two of our samples, which was expected as these two samples (07PB1887 and 10PB6041) came from the same patient (Fig. 3).

### Quality control of normalization procedure

**Fig. 4:**
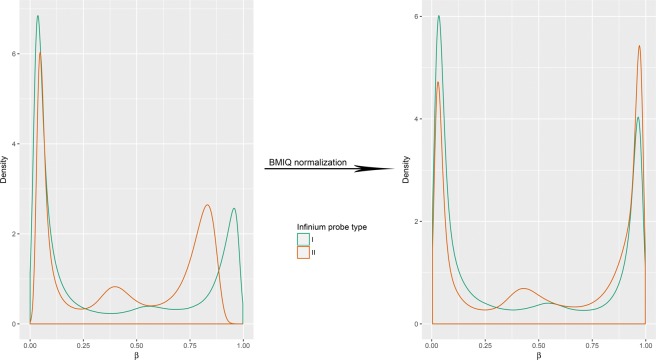
Density plots of the β-values distribution for the Infinium I and II probes before and after background subtraction and beta-mixture quantile normalization (BMIQ).

Preprocessing of the raw data was performed using R/Bioconductor with the RnBeads package^24^. Probes known to be cross-reactive (43 230) or overlapping known single-nucleotide polymorphisms (SNPs; 8 704)^25^, as well as probes giving unreliable measurements (884 as determined by the Greedycut algorithm) were excluded from analysis. The data from the remaining 432 759 probes were subjected to background subtraction using the Noob method^26^ and beta-mixture quantile normalization (BMIQ)^27^. This normalization strategy successfully mitigated the inherent bias in β-value distributions between the two different types of probes (Infinium I and II) that are present on the HumanMethylation450 BeadChip^30^, as shown in Fig. 4. In a subsequent step, probes of non-CpG context (1 251), probes binding to sequences on sex chromosomes (9 917) and probes with standard deviation <0.005 (69 867) were filtered out. Thus, the data obtained by the remaining 351 724 probes qualified for downstream analysis.

### Check for batch effects

**Fig. 5:**
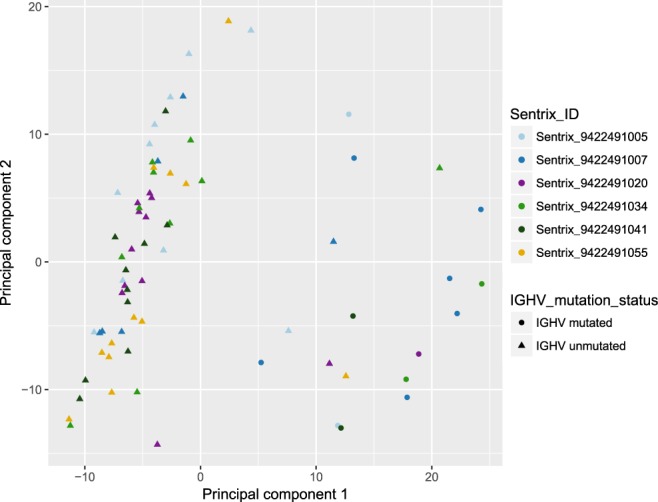
Principal component analysis (PCA) showing grouping of the 72 samples based on the 10000 most variable CpGs within the dataset. Samples are coloured according to the BeadChip (Sentrix_ID) that they were applied on. Samples of any given colour (batch) do not form separate clusters, whereas principal component 1 distinguishes M-CLL from U-CLL patients (P = 7.15 × 10^−8^, Wilcoxon rank sum test).

Dimension reduction techniques are a powerful way of visualizing associations between different variables and global trends in DNA methylation data^24^. Applying PCA on the 10000 most variable CpGs in our data did not result in visible grouping of samples according to the BeadChip that they were applied on (Fig. 5). The lack of batch effects was further verified by Kruskal-Wallis one-way analysis of variance taking into account the first 8 primary components (Table 2).

**Table 2:** Table of p-values for associations of the first 8 principal components with *IGHV* mutation status as an intrinsic characteristic of the samples or with the BeadChip that the samples were applied on as an extrinsic variable (batch).

Trait	Principal component							
	1	2	3	4	5	6	7	8
BeadChip number (Sentrix_ID)*	0.15	0.26	0.21	0.22	0.24	0.68	0.65	0.27
*IGHV* mutation status^^^	7.15 × 10^–8^	0.08	0.15	0.73	0.78	0.65	0.33	0.62

*P-values from a Kruskal-Wallis one-way analysis of variance.

^^^P-values from a two-sided Wilcoxon rank sum test.

### Biological validation of the DNA methylation data

**Fig. 6:**
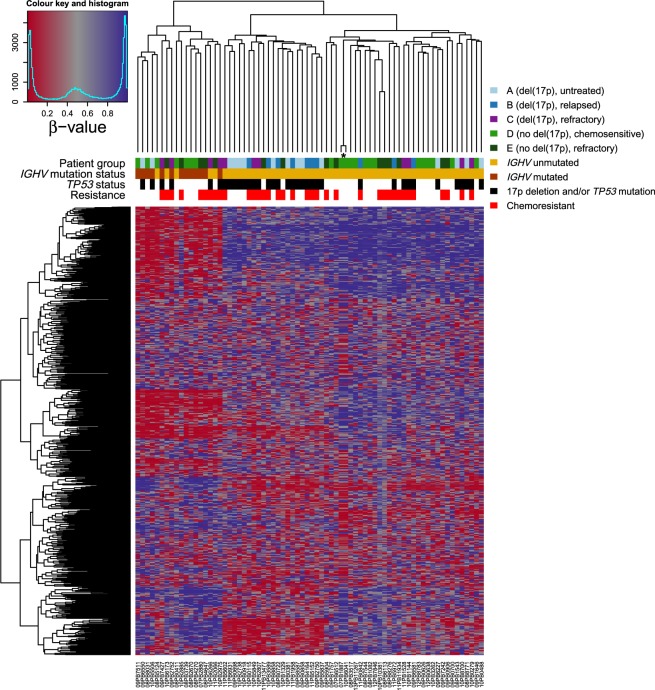
Unsupervised hierarchical clustering of the 72 CLL samples based on the β-values of the 5000 most variable CpG sites. Clustering was based on Manhattan distance with complete linkage. Columns represent samples and rows CpGs. Two of the samples originate from the same patient (see the main text) and are marked by an asterisk. The high similarity of DNA methylation patterns between them affirms the reproducibility of the methodology.

A technically sound dataset would allow confirmation of known facts. Using our dataset, we could replicate the finding that M-CLL and U-CLL are associated with distinct DNA methylation profiles^7^. In addition to the PCA in Fig. 5 and Table 2, we performed unsupervised hierarchical clustering using the 5000 most variable CpG sites. The resulting dendrograms and heatmap of β-values are presented in Fig. 6. In both analyses, samples were well separated according to IGHV mutation status, with the bigger cluster consisting only of U-CLL cases and the smaller cluster comprising all M-CLL cases plus five U-CLL cases, three of which had a considerable level of IGHV hypermutation (98.2–98.3% homology to germline; see also Online-only Table 1), thus possibly belonging to the intermediate CLL group as defined by Kulis *et al*.^7^.

The highly similar DNA methylation profiles of the two serial samples from one patient (Fig. 6) demonstrated the reproducibility and robustness of the whole analytical procedure (the two samples were hybridized to different BeadChips) and in addition supported previous observations that evolution of DNA methylation in CLL is limited to cases that acquire high-risk genetic alterations^31^.

## Usage notes

In our exploratory analysis, we could not observe obvious clustering of samples based on patients’ sensitivity or resistance to chemotherapy (Figs. 5 and 6). Accordingly, a differential methylation test using the limma method with the threshold for difference of β-values set to 0.1 and the false discovery rate set to 0.05 could not find any differentially methylated CpGs between chemoresistant and untreated/chemosensitive patients, neither in the subgroup of patients with del(17p)/*TP53*mut (groups B and C vs. group A), nor in the subgroup without these aberrations (group E vs. group D). Nevertheless, as multiple testing correction inflates the type II error rate considerably, we cannot exclude that some of the tested CpGs have truly different methylation between the groups and a causative role in chemoresistance development. In this regard, our dataset can be a valuable resource for conducting hypothesis-driven research addressing questions of chemoresistance in CLL.

Additionally, thanks to the rich annotation of the samples, our dataset can be used to explore associations of DNA methylation with other markers of prognostic or predictive value in CLL, e.g. presence of specific chromosome aberrations (see Table 1 and Online-only Table 1). Such analyses should be performed with the necessary caution and subsequent validation of the findings in an independent clinical cohort^32^. Conversely, our dataset can serve as the validation dataset for findings originating from other clinical cohorts.

DNA methylation is highly informative when analyzed together with additional layers of the epigenomic regulatory landscape. That is why we recommend that any CpGs of interest found to be differentially methylated between groups be analyzed in the context of published whole-genome maps of histone modifications, chromatin accessibility and chromatin states of CLL and normal B cells^33^. Data from the ENCODE project can also be useful if parallels with a broader variety of cell types are sought^34,35^. Additional insights can be gained if tools like HOMER are used to identify transcription factor recognition motifs around CpGs of interest.

### Supplementary information

Supplementary File 1

## Online-only Tables

**Online-only Table 1:** Clinical and molecular characteristics of CLL patients included in the study.

Experimental group	Sample ID	Patient sex^	Predicted sex^	Patient age, years	Treated	Response to therapy	IGHV mutation status (% homology)	del13q14	Nuclei with del13q14 (%)	+12p	Nuclei with trisomy 12 (%)	del14q32	Nuclei with del14q32 (%)	del11q22	Nuclei with del11q22 (%)	del17p13	Nuclei with del17p13 (%)	TP53 mutation	TP53 mutation type	TP53 variant allele frequency (%)	Protocol 1	Protocol 2	Protocol 3	Protocol 4	Protocol 5	Output files	Sample name in GEO	Alternative sample title in GEO	Protocol 6	Final processed output
A (del(17p), untreated)	09PB8587	NK	Female	68	No	NA	100	No	4.5	No	0.0	No	1.5	No	1.50	Yes	18.00	c.830G>T	missense	5	Isolation of peripheral blood mononuclear cells (PBMCs) from blood samples via density gradient centrifugation (Pancoll human)	Purification of CD19-positive cell fraction using CD19 MicroBeads	Genomic DNA extraction (Qiagen AllPrep kit)	Bisulfite DNA conversion, whole-genome amplification, fragmentation and hybridization to Illumina Infinium HumanMethylation450 BeadChips (standard Illumina protocols)	Single-base extension, staining and scanning (standard Illumina protocols)	9422491005_R01C01_Grn.idat & 9422491005_R01C01_Red.idat	GSM4056697	CLL_A_del17p_untreated_1	Raw data filtering, background subtraction (Noob) and normalization (BMIQ)	Data matrix of beta-values
A (del(17p), untreated)	10PB5506	NK	Female	71	No	NA	82.62	Yes	86.50	No	4.5	No	2.0	No	4.50	Yes	56.50	c.527G>C; 528C>T	missense	50	Isolation of peripheral blood mononuclear cells (PBMCs) from blood samples via density gradient centrifugation (Pancoll human)	Purification of CD19-positive cell fraction using CD19 MicroBeads	Genomic DNA extraction (Qiagen AllPrep kit)	Bisulfite DNA conversion, whole-genome amplification, fragmentation and hybridization to Illumina Infinium HumanMethylation450 BeadChips (standard Illumina protocols)	Single-base extension, staining and scanning (standard Illumina protocols)	9422491007_R05C02_Grn.idat & 9422491007_R05C02_Red.idat	GSM4056718	CLL_A_del17p_untreated_6	Raw data filtering, background subtraction (Noob) and normalization (BMIQ)	Data matrix of beta-values
A (del(17p), untreated)	10PB8858	NK	Female	77	No	NA	100	Yes	87.00	No	1.5	No	2.0	No	2.50	Yes	89.00	c.731G>T	missense	90	Isolation of peripheral blood mononuclear cells (PBMCs) from blood samples via density gradient centrifugation (Pancoll human)	Purification of CD19-positive cell fraction using CD19 MicroBeads	Genomic DNA extraction (Qiagen AllPrep kit)	Bisulfite DNA conversion, whole-genome amplification, fragmentation and hybridization to Illumina Infinium HumanMethylation450 BeadChips (standard Illumina protocols)	Single-base extension, staining and scanning (standard Illumina protocols)	9422491034_R05C02_Grn.idat & 9422491034_R05C02_Red.idat	GSM4056742	CLL_A_del17p_untreated_10	Raw data filtering, background subtraction (Noob) and normalization (BMIQ)	Data matrix of beta-values
A (del(17p), untreated)	09PB6227	NK	Male	61	No	NA	100	No	1.5	No	2.00	No	1.50	Yes	14.50	Yes	19.00	none detected	NA	0	Isolation of peripheral blood mononuclear cells (PBMCs) from blood samples via density gradient centrifugation (Pancoll human)	Purification of CD19-positive cell fraction using CD19 MicroBeads	Genomic DNA extraction (Qiagen AllPrep kit)	Bisulfite DNA conversion, whole-genome amplification, fragmentation and hybridization to Illumina Infinium HumanMethylation450 BeadChips (standard Illumina protocols)	Single-base extension, staining and scanning (standard Illumina protocols)	9422491005_R06C01_Grn.idat & 9422491005_R06C01_Red.idat	GSM4056707	CLL_A_del17p_untreated_3	Raw data filtering, background subtraction (Noob) and normalization (BMIQ)	Data matrix of beta-values
A (del(17p), untreated)	09PB0858	NK	Female	66	No	NA	100	Yes	24.50	No	2.0	No	5.5	No	1.00	Yes	94.50	none detected	NA	0	Isolation of peripheral blood mononuclear cells (PBMCs) from blood samples via density gradient centrifugation (Pancoll human)	Purification of CD19-positive cell fraction using CD19 MicroBeads	Genomic DNA extraction (Qiagen AllPrep kit)	Bisulfite DNA conversion, whole-genome amplification, fragmentation and hybridization to Illumina Infinium HumanMethylation450 BeadChips (standard Illumina protocols)	Single-base extension, staining and scanning (standard Illumina protocols)	9422491020_R01C01_Grn.idat & 9422491020_R01C01_Red.idat	GSM4056721	CLL_A_del17p_untreated_7	Raw data filtering, background subtraction (Noob) and normalization (BMIQ)	Data matrix of beta-values
A (del(17p), untreated)	08PB1543	Female	Female	49	No	NA	99.5	No	5.5	No	0.5	No	1.5	No	4.00	Yes	85.50	c.734G>A	missense	90	Isolation of peripheral blood mononuclear cells (PBMCs) from blood samples via density gradient centrifugation (Pancoll human)	Purification of CD19-positive cell fraction using CD19 MicroBeads	Genomic DNA extraction (Qiagen AllPrep kit)	Bisulfite DNA conversion, whole-genome amplification, fragmentation and hybridization to Illumina Infinium HumanMethylation450 BeadChips (standard Illumina protocols)	Single-base extension, staining and scanning (standard Illumina protocols)	9422491041_R03C01_Grn.idat & 9422491041_R03C01_Red.idat	GSM4056749	CLL_A_del17p_untreated_13	Raw data filtering, background subtraction (Noob) and normalization (BMIQ)	Data matrix of beta-values
A (del(17p), untreated)	10PB0381	NK	Female	61	No	NA	100	No	2.0	No	2.50	No	2.50	Yes	75.00	Yes	84.50	c.722C>T	missense	50	Isolation of peripheral blood mononuclear cells (PBMCs) from blood samples via density gradient centrifugation (Pancoll human)	Purification of CD19-positive cell fraction using CD19 MicroBeads	Genomic DNA extraction (Qiagen AllPrep kit)	Bisulfite DNA conversion, whole-genome amplification, fragmentation and hybridization to Illumina Infinium HumanMethylation450 BeadChips (standard Illumina protocols)	Single-base extension, staining and scanning (standard Illumina protocols)	9422491005_R05C02_Grn.idat & 9422491005_R05C02_Red.idat	GSM4056706	CLL_A_del17p_untreated_2	Raw data filtering, background subtraction (Noob) and normalization (BMIQ)	Data matrix of beta-values
A (del(17p), untreated)	10PB0918	NK	Male	51	No	NA	99.53	Yes	94.00	No	5.0	No	3.0	No	1.50	Yes	98.00	c.742C>T	missense	100	Isolation of peripheral blood mononuclear cells (PBMCs) from blood samples via density gradient centrifugation (Pancoll human)	Purification of CD19-positive cell fraction using CD19 MicroBeads	Genomic DNA extraction (Qiagen AllPrep kit)	Bisulfite DNA conversion, whole-genome amplification, fragmentation and hybridization to Illumina Infinium HumanMethylation450 BeadChips (standard Illumina protocols)	Single-base extension, staining and scanning (standard Illumina protocols)	9422491020_R06C01_Grn.idat & 9422491020_R06C01_Red.idat	GSM4056731	CLL_A_del17p_untreated_8	Raw data filtering, background subtraction (Noob) and normalization (BMIQ)	Data matrix of beta-values
A (del(17p), untreated)	10PB0488	NK	Male	51	No	NA	99.52	Yes	84.00	No	0.5	No	2.0	No	3.00	Yes	92.00	none detected	NA	0	Isolation of peripheral blood mononuclear cells (PBMCs) from blood samples via density gradient centrifugation (Pancoll human)	Purification of CD19-positive cell fraction using CD19 MicroBeads	Genomic DNA extraction (Qiagen AllPrep kit)	Bisulfite DNA conversion, whole-genome amplification, fragmentation and hybridization to Illumina Infinium HumanMethylation450 BeadChips (standard Illumina protocols)	Single-base extension, staining and scanning (standard Illumina protocols)	9422491041_R02C02_Grn.idat & 9422491041_R02C02_Red.idat	GSM4056748	CLL_A_del17p_untreated_12	Raw data filtering, background subtraction (Noob) and normalization (BMIQ)	Data matrix of beta-values
A (del(17p), untreated)	09PB6550	Female	Female	51	No	NA	92.64	Yes	89.50	No	1.0	No	3.0	No	2.00	Yes	23.00	none detected	NA	0	Isolation of peripheral blood mononuclear cells (PBMCs) from blood samples via density gradient centrifugation (Pancoll human)	Purification of CD19-positive cell fraction using CD19 MicroBeads	Genomic DNA extraction (Qiagen AllPrep kit)	Bisulfite DNA conversion, whole-genome amplification, fragmentation and hybridization to Illumina Infinium HumanMethylation450 BeadChips (standard Illumina protocols)	Single-base extension, staining and scanning (standard Illumina protocols)	9422491007_R03C01_Grn.idat & 9422491007_R03C01_Red.idat	GSM4056713	CLL_A_del17p_untreated_5	Raw data filtering, background subtraction (Noob) and normalization (BMIQ)	Data matrix of beta-values
A (del(17p), untreated)	08PB8771	Male	Male	69	No	NA	100	Yes	86.0	No	1.0	No	1.0	Yes	90.50	Yes	92.50	c.329G>T	missense	80	Isolation of peripheral blood mononuclear cells (PBMCs) from blood samples via density gradient centrifugation (Pancoll human)	Purification of CD19-positive cell fraction using CD19 MicroBeads	Genomic DNA extraction (Qiagen AllPrep kit)	Bisulfite DNA conversion, whole-genome amplification, fragmentation and hybridization to Illumina Infinium HumanMethylation450 BeadChips (standard Illumina protocols)	Single-base extension, staining and scanning (standard Illumina protocols)	9422491034_R01C01_Grn.idat & 9422491034_R01C01_Red.idat	GSM4056733	CLL_A_del17p_untreated_9	Raw data filtering, background subtraction (Noob) and normalization (BMIQ)	Data matrix of beta-values
A (del(17p), untreated)	09PB5837	NK	Male	65	No	NA	99.5	No	3.0	Yes	53.5	No	2.5	No	0.50	Yes	59.50	c.413C>T	missense	45	Isolation of peripheral blood mononuclear cells (PBMCs) from blood samples via density gradient centrifugation (Pancoll human)	Purification of CD19-positive cell fraction using CD19 MicroBeads	Genomic DNA extraction (Qiagen AllPrep kit)	Bisulfite DNA conversion, whole-genome amplification, fragmentation and hybridization to Illumina Infinium HumanMethylation450 BeadChips (standard Illumina protocols)	Single-base extension, staining and scanning (standard Illumina protocols)	9422491055_R01C01_Grn.idat & 9422491055_R01C01_Red.idat	GSM4056757	CLL_A_del17p_untreated_14	Raw data filtering, background subtraction (Noob) and normalization (BMIQ)	Data matrix of beta-values
A (del(17p), untreated)	10PB2738	Male	Male	68	No	NA	99.52	Yes	30.0	Yes	58.0	No	1.0	No	3.50	Yes	92.50	none detected	NA	0	Isolation of peripheral blood mononuclear cells (PBMCs) from blood samples via density gradient centrifugation (Pancoll human)	Purification of CD19-positive cell fraction using CD19 MicroBeads	Genomic DNA extraction (Qiagen AllPrep kit)	Bisulfite DNA conversion, whole-genome amplification, fragmentation and hybridization to Illumina Infinium HumanMethylation450 BeadChips (standard Illumina protocols)	Single-base extension, staining and scanning (standard Illumina protocols)	9422491007_R02C02_Grn.idat & 9422491007_R02C02_Red.idat	GSM4056712	CLL_A_del17p_untreated_4	Raw data filtering, background subtraction (Noob) and normalization (BMIQ)	Data matrix of beta-values
A (del(17p), untreated)	11PB6931	NK	Female	67	No	NA	99.05	No	3.5	No	0.00	Yes	86.0	No	0.50	Yes	64.00	c.743G>A	missense	40	Isolation of peripheral blood mononuclear cells (PBMCs) from blood samples via density gradient centrifugation (Pancoll human)	Purification of CD19-positive cell fraction using CD19 MicroBeads	Genomic DNA extraction (Qiagen AllPrep kit)	Bisulfite DNA conversion, whole-genome amplification, fragmentation and hybridization to Illumina Infinium HumanMethylation450 BeadChips (standard Illumina protocols)	Single-base extension, staining and scanning (standard Illumina protocols)	9422491034_R06C01_Grn.idat & 9422491034_R06C01_Red.idat	GSM4056743	CLL_A_del17p_untreated_11	Raw data filtering, background subtraction (Noob) and normalization (BMIQ)	Data matrix of beta-values
A (del(17p), untreated)	09PB0698	Female	Female	72	No	NA	100	Yes	53.0	Yes	16.5	No	5.0	No	3.50	Yes	73.50	c.809T>G	missense	80	Isolation of peripheral blood mononuclear cells (PBMCs) from blood samples via density gradient centrifugation (Pancoll human)	Purification of CD19-positive cell fraction using CD19 MicroBeads	Genomic DNA extraction (Qiagen AllPrep kit)	Bisulfite DNA conversion, whole-genome amplification, fragmentation and hybridization to Illumina Infinium HumanMethylation450 BeadChips (standard Illumina protocols)	Single-base extension, staining and scanning (standard Illumina protocols)	9422491055_R06C01_Grn.idat & 9422491055_R06C01_Red.idat	GSM4056767	CLL_A_del17p_untreated_15	Raw data filtering, background subtraction (Noob) and normalization (BMIQ)	Data matrix of beta-values
B (del(17p), relapsed)	11PB5152	NK	Male	68	Yes	Relapsed	100	Yes	8.5	No	2.0	No	0.0	No	1.50	Yes	24.00	c.536A>G; c.733G>T	missense; missense	50; 10	Isolation of peripheral blood mononuclear cells (PBMCs) from blood samples via density gradient centrifugation (Pancoll human)	Purification of CD19-positive cell fraction using CD19 MicroBeads	Genomic DNA extraction (Qiagen AllPrep kit)	Bisulfite DNA conversion, whole-genome amplification, fragmentation and hybridization to Illumina Infinium HumanMethylation450 BeadChips (standard Illumina protocols)	Single-base extension, staining and scanning (standard Illumina protocols)	9422491005_R02C01_Grn.idat & 9422491005_R02C01_Red.idat	GSM4056699	CLL_B_del17p_relapsed_2	Raw data filtering, background subtraction (Noob) and normalization (BMIQ)	Data matrix of beta-values
B (del(17p), relapsed)	09PB6581	NK	Male	59	Yes	Relapsed	100	Yes	30.5	No	1.5	No	1.0	No	3.50	Yes	87.00	c.794T>C	missense	60	Isolation of peripheral blood mononuclear cells (PBMCs) from blood samples via density gradient centrifugation (Pancoll human)	Purification of CD19-positive cell fraction using CD19 MicroBeads	Genomic DNA extraction (Qiagen AllPrep kit)	Bisulfite DNA conversion, whole-genome amplification, fragmentation and hybridization to Illumina Infinium HumanMethylation450 BeadChips (standard Illumina protocols)	Single-base extension, staining and scanning (standard Illumina protocols)	9422491020_R01C02_Grn.idat & 9422491020_R01C02_Red.idat	GSM4056722	CLL_B_del17p_relapsed_4	Raw data filtering, background subtraction (Noob) and normalization (BMIQ)	Data matrix of beta-values
B (del(17p), relapsed)	09PB2750	NK	Male	54	Yes	Relapsed	99.65	No	2.0	Yes	74.0	No	6.0	No	3.00	Yes	15.50	c.535C>T	missense	60	Isolation of peripheral blood mononuclear cells (PBMCs) from blood samples via density gradient centrifugation (Pancoll human)	Purification of CD19-positive cell fraction using CD19 MicroBeads	Genomic DNA extraction (Qiagen AllPrep kit)	Bisulfite DNA conversion, whole-genome amplification, fragmentation and hybridization to Illumina Infinium HumanMethylation450 BeadChips (standard Illumina protocols)	Single-base extension, staining and scanning (standard Illumina protocols)	9422491041_R04C01_Grn.idat & 9422491041_R04C01_Red.idat	GSM4056751	CLL_B_del17p_relapsed_8	Raw data filtering, background subtraction (Noob) and normalization (BMIQ)	Data matrix of beta-values
B (del(17p), relapsed)	10PB0115	NK	Female	60	Yes	Relapsed	99.1	No	5.0	No	0.5	Yes	61.0	Yes	91.00	Yes	64.50	c.632C>T; c.675–683del9bp	missense; in frame deletion	55; 5	Isolation of peripheral blood mononuclear cells (PBMCs) from blood samples via density gradient centrifugation (Pancoll human)	Purification of CD19-positive cell fraction using CD19 MicroBeads	Genomic DNA extraction (Qiagen AllPrep kit)	Bisulfite DNA conversion, whole-genome amplification, fragmentation and hybridization to Illumina Infinium HumanMethylation450 BeadChips (standard Illumina protocols)	Single-base extension, staining and scanning (standard Illumina protocols)	9422491005_R01C02_Grn.idat & 9422491005_R01C02_Red.idat	GSM4056698	CLL_B_del17p_relapsed_1	Raw data filtering, background subtraction (Noob) and normalization (BMIQ)	Data matrix of beta-values
B (del(17p), relapsed)	10PB3589	NK	Male	64	Yes	Relapsed	100	Yes	53.5	No	3.5	Yes	87.0	No	4.00	Yes	91.50	c.652–654del3bp	in frame deletion	15	Isolation of peripheral blood mononuclear cells (PBMCs) from blood samples via density gradient centrifugation (Pancoll human)	Purification of CD19-positive cell fraction using CD19 MicroBeads	Genomic DNA extraction (Qiagen AllPrep kit)	Bisulfite DNA conversion, whole-genome amplification, fragmentation and hybridization to Illumina Infinium HumanMethylation450 BeadChips (standard Illumina protocols)	Single-base extension, staining and scanning (standard Illumina protocols)	9422491034_R02C01_Grn.idat & 9422491034_R02C01_Red.idat	GSM4056735	CLL_B_del17p_relapsed_7	Raw data filtering, background subtraction (Noob) and normalization (BMIQ)	Data matrix of beta-values
B (del(17p), relapsed)	11PB1958	NK	Male	64	Yes	Relapsed	100	Yes	42.5	No	1.0	No	2.0	No	3.00	Yes	67.00	c.584T>C; c.673-2A>C; c.842A>T	missence; splice site; missense	30; 20; 20	Isolation of peripheral blood mononuclear cells (PBMCs) from blood samples via density gradient centrifugation (Pancoll human)	Purification of CD19-positive cell fraction using CD19 MicroBeads	Genomic DNA extraction (Qiagen AllPrep kit)	Bisulfite DNA conversion, whole-genome amplification, fragmentation and hybridization to Illumina Infinium HumanMethylation450 BeadChips (standard Illumina protocols)	Single-base extension, staining and scanning (standard Illumina protocols)	9422491055_R02C01_Grn.idat & 9422491055_R02C01_Red.idat	GSM4056759	CLL_B_del17p_relapsed_9	Raw data filtering, background subtraction (Noob) and normalization (BMIQ)	Data matrix of beta-values
B (del(17p), relapsed)	11PB5972	NK	Male	67	Yes	Relapsed	100	Yes	88.5	No	1.5	No	1.5	Yes	14.50	Yes	20.50	c.742C>T	missense	30	Isolation of peripheral blood mononuclear cells (PBMCs) from blood samples via density gradient centrifugation (Pancoll human)	Purification of CD19-positive cell fraction using CD19 MicroBeads	Genomic DNA extraction (Qiagen AllPrep kit)	Bisulfite DNA conversion, whole-genome amplification, fragmentation and hybridization to Illumina Infinium HumanMethylation450 BeadChips (standard Illumina protocols)	Single-base extension, staining and scanning (standard Illumina protocols)	9422491007_R04C01_Grn.idat & 9422491007_R04C01_Red.idat	GSM4056715	CLL_B_del17p_relapsed_3	Raw data filtering, background subtraction (Noob) and normalization (BMIQ)	Data matrix of beta-values
B (del(17p), relapsed)	08PB0722	Male	Male	58	Yes	Relapsed	100	No	4.0	No	0.0	No	2.0	Yes	89.50	Yes	88.50	c.452C>A	missense	90	Isolation of peripheral blood mononuclear cells (PBMCs) from blood samples via density gradient centrifugation (Pancoll human)	Purification of CD19-positive cell fraction using CD19 MicroBeads	Genomic DNA extraction (Qiagen AllPrep kit)	Bisulfite DNA conversion, whole-genome amplification, fragmentation and hybridization to Illumina Infinium HumanMethylation450 BeadChips (standard Illumina protocols)	Single-base extension, staining and scanning (standard Illumina protocols)	9422491034_R01C02_Grn.idat & 9422491034_R01C02_Red.idat	GSM4056734	CLL_B_del17p_relapsed_6	Raw data filtering, background subtraction (Noob) and normalization (BMIQ)	Data matrix of beta-values
B (del(17p), relapsed)	11PB0842	NK	Female	67	Yes	Relapsed	100	Yes	88.0	No	0.5	No	4.5	No	2.50	Yes	13.50	none detected	NA	0	Isolation of peripheral blood mononuclear cells (PBMCs) from blood samples via density gradient centrifugation (Pancoll human)	Purification of CD19-positive cell fraction using CD19 MicroBeads	Genomic DNA extraction (Qiagen AllPrep kit)	Bisulfite DNA conversion, whole-genome amplification, fragmentation and hybridization to Illumina Infinium HumanMethylation450 BeadChips (standard Illumina protocols)	Single-base extension, staining and scanning (standard Illumina protocols)	9422491055_R02C02_Grn.idat & 9422491055_R02C02_Red.idat	GSM4056760	CLL_B_del17p_relapsed_10	Raw data filtering, background subtraction (Noob) and normalization (BMIQ)	Data matrix of beta-values
B (del(17p), relapsed)	09PB4164	Male	Male	53	Yes	Relapsed	100	No	3.5	No	1.00	No	2.50	No	4.50	Yes	83.00	c.557-559+5del8bp	in frame deletion	20	Isolation of peripheral blood mononuclear cells (PBMCs) from blood samples via density gradient centrifugation (Pancoll human)	Purification of CD19-positive cell fraction using CD19 MicroBeads	Genomic DNA extraction (Qiagen AllPrep kit)	Bisulfite DNA conversion, whole-genome amplification, fragmentation and hybridization to Illumina Infinium HumanMethylation450 BeadChips (standard Illumina protocols)	Single-base extension, staining and scanning (standard Illumina protocols)	9422491020_R02C01_Grn.idat & 9422491020_R02C01_Red.idat	GSM4056723	CLL_B_del17p_relapsed_5	Raw data filtering, background subtraction (Noob) and normalization (BMIQ)	Data matrix of beta-values
C (del(17p), refractory)	09PB7427	NK	Male	73	Yes	Refractory	94.6	Yes	74.5	Yes	65.5	No	3.5	No	0.50	Yes	86.50	c.394A>G	missense	60	Isolation of peripheral blood mononuclear cells (PBMCs) from blood samples via density gradient centrifugation (Pancoll human)	Purification of CD19-positive cell fraction using CD19 MicroBeads	Genomic DNA extraction (Qiagen AllPrep kit)	Bisulfite DNA conversion, whole-genome amplification, fragmentation and hybridization to Illumina Infinium HumanMethylation450 BeadChips (standard Illumina protocols)	Single-base extension, staining and scanning (standard Illumina protocols)	9422491005_R03C01_Grn.idat & 9422491005_R03C01_Red.idat	GSM4056701	CLL_C_del17p_refractory_2	Raw data filtering, background subtraction (Noob) and normalization (BMIQ)	Data matrix of beta-values
C (del(17p), refractory)	11PB1528	NK	Male	76	Yes	Refractory	100	No	8.5	No	4.50	No	1.50	No	2.50	Yes	52.00	c.785G>T	missense	50	Isolation of peripheral blood mononuclear cells (PBMCs) from blood samples via density gradient centrifugation (Pancoll human)	Purification of CD19-positive cell fraction using CD19 MicroBeads	Genomic DNA extraction (Qiagen AllPrep kit)	Bisulfite DNA conversion, whole-genome amplification, fragmentation and hybridization to Illumina Infinium HumanMethylation450 BeadChips (standard Illumina protocols)	Single-base extension, staining and scanning (standard Illumina protocols)	9422491020_R02C02_Grn.idat & 9422491020_R02C02_Red.idat	GSM4056724	CLL_C_del17p_refractory_4	Raw data filtering, background subtraction (Noob) and normalization (BMIQ)	Data matrix of beta-values
C (del(17p), refractory)	10PB8700	NK	Male	64	Yes	Refractory	100	Yes	95.5	No	1.5	No	4.0	No	6.50	Yes	98.50	c.429_430ins3bp	in frame insertion	100	Isolation of peripheral blood mononuclear cells (PBMCs) from blood samples via density gradient centrifugation (Pancoll human)	Purification of CD19-positive cell fraction using CD19 MicroBeads	Genomic DNA extraction (Qiagen AllPrep kit)	Bisulfite DNA conversion, whole-genome amplification, fragmentation and hybridization to Illumina Infinium HumanMethylation450 BeadChips (standard Illumina protocols)	Single-base extension, staining and scanning (standard Illumina protocols)	9422491041_R05C02_Grn.idat & 9422491041_R05C02_Red.idat	GSM4056754	CLL_C_del17p_refractory_9	Raw data filtering, background subtraction (Noob) and normalization (BMIQ)	Data matrix of beta-values
C (del(17p), refractory)	10PB2807	Female	Female	75	Yes	Refractory	100	Yes	25.5	No	1.0	No	0.5	No	2.00	Yes	94.00	c.733G>T	missense	90	Isolation of peripheral blood mononuclear cells (PBMCs) from blood samples via density gradient centrifugation (Pancoll human)	Purification of CD19-positive cell fraction using CD19 MicroBeads	Genomic DNA extraction (Qiagen AllPrep kit)	Bisulfite DNA conversion, whole-genome amplification, fragmentation and hybridization to Illumina Infinium HumanMethylation450 BeadChips (standard Illumina protocols)	Single-base extension, staining and scanning (standard Illumina protocols)	9422491005_R02C02_Grn.idat & 9422491005_R02C02_Red.idat	GSM4056700	CLL_C_del17p_refractory_1	Raw data filtering, background subtraction (Noob) and normalization (BMIQ)	Data matrix of beta-values
C (del(17p), refractory)	10PB1144	NK	Male	76	Yes	Refractory	100	Yes	88.5	No	1.5	No	1.0	No	3.50	Yes	30.50	c.734G>T	missense	20	Isolation of peripheral blood mononuclear cells (PBMCs) from blood samples via density gradient centrifugation (Pancoll human)	Purification of CD19-positive cell fraction using CD19 MicroBeads	Genomic DNA extraction (Qiagen AllPrep kit)	Bisulfite DNA conversion, whole-genome amplification, fragmentation and hybridization to Illumina Infinium HumanMethylation450 BeadChips (standard Illumina protocols)	Single-base extension, staining and scanning (standard Illumina protocols)	9422491034_R03C01_Grn.idat & 9422491034_R03C01_Red.idat	GSM4056737	CLL_C_del17p_refractory_7	Raw data filtering, background subtraction (Noob) and normalization (BMIQ)	Data matrix of beta-values
C (del(17p), refractory)	10PB5849	NK	Female	64	Yes	Refractory	99.03	No	5.0	Yes	60.0	No	1.5	No	2.00	Yes	90.00	c.672+1G>T	splice site	85	Isolation of peripheral blood mononuclear cells (PBMCs) from blood samples via density gradient centrifugation (Pancoll human)	Purification of CD19-positive cell fraction using CD19 MicroBeads	Genomic DNA extraction (Qiagen AllPrep kit)	Bisulfite DNA conversion, whole-genome amplification, fragmentation and hybridization to Illumina Infinium HumanMethylation450 BeadChips (standard Illumina protocols)	Single-base extension, staining and scanning (standard Illumina protocols)	9422491055_R03C01_Grn.idat & 9422491055_R03C01_Red.idat	GSM4056761	CLL_C_del17p_refractory_10	Raw data filtering, background subtraction (Noob) and normalization (BMIQ)	Data matrix of beta-values
C (del(17p), refractory)	09PB2752	NK	Male	54	Yes	Refractory	97.65	Yes	88.0	No	1.5	Yes	63.5	No	5.00	Yes	43.50	c.375+1G>T; c.523C>G	splice site; missense	45; 50	Isolation of peripheral blood mononuclear cells (PBMCs) from blood samples via density gradient centrifugation (Pancoll human)	Purification of CD19-positive cell fraction using CD19 MicroBeads	Genomic DNA extraction (Qiagen AllPrep kit)	Bisulfite DNA conversion, whole-genome amplification, fragmentation and hybridization to Illumina Infinium HumanMethylation450 BeadChips (standard Illumina protocols)	Single-base extension, staining and scanning (standard Illumina protocols)	9422491007_R05C01_Grn.idat & 9422491007_R05C01_Red.idat	GSM4056717	CLL_C_del17p_refractory_3	Raw data filtering, background subtraction (Noob) and normalization (BMIQ)	Data matrix of beta-values
C (del(17p), refractory)	11PB5602	NK	Female	67	Yes	Refractory	100	No	1.5	Yes	69.5	No	2.5	No	3.00	Yes	77.50	c.176_188del13bp; c.669_672+10del14bp	frameshift; frameshift	5; 5	Isolation of peripheral blood mononuclear cells (PBMCs) from blood samples via density gradient centrifugation (Pancoll human)	Purification of CD19-positive cell fraction using CD19 MicroBeads	Genomic DNA extraction (Qiagen AllPrep kit)	Bisulfite DNA conversion, whole-genome amplification, fragmentation and hybridization to Illumina Infinium HumanMethylation450 BeadChips (standard Illumina protocols)	Single-base extension, staining and scanning (standard Illumina protocols)	9422491034_R02C02_Grn.idat & 9422491034_R02C02_Red.idat	GSM4056736	CLL_C_del17p_refractory_6	Raw data filtering, background subtraction (Noob) and normalization (BMIQ)	Data matrix of beta-values
C (del(17p), refractory)	10PB6266	Male	Male	60	Yes	Refractory	98.3	Yes	92.0	No	3.0	No	2.5	Yes	91.50	No	3.00	c.386ins1bp	frameshift	90	Isolation of peripheral blood mononuclear cells (PBMCs) from blood samples via density gradient centrifugation (Pancoll human)	Purification of CD19-positive cell fraction using CD19 MicroBeads	Genomic DNA extraction (Qiagen AllPrep kit)	Bisulfite DNA conversion, whole-genome amplification, fragmentation and hybridization to Illumina Infinium HumanMethylation450 BeadChips (standard Illumina protocols)	Single-base extension, staining and scanning (standard Illumina protocols)	9422491055_R04C02_Grn.idat & 9422491055_R04C02_Red.idat	GSM4056764	CLL_C_del17p_refractory_11	Raw data filtering, background subtraction (Noob) and normalization (BMIQ)	Data matrix of beta-values
C (del(17p), refractory)	10PB0279	NK	Male	72	Yes	Refractory	100	Yes	73.0	No	1.0	No	3.5	Yes	95.50	Yes	94.50	c.277–277delC; c.594–599del6bp	frameshift; in frame deletion	90; 10	Isolation of peripheral blood mononuclear cells (PBMCs) from blood samples via density gradient centrifugation (Pancoll human)	Purification of CD19-positive cell fraction using CD19 MicroBeads	Genomic DNA extraction (Qiagen AllPrep kit)	Bisulfite DNA conversion, whole-genome amplification, fragmentation and hybridization to Illumina Infinium HumanMethylation450 BeadChips (standard Illumina protocols)	Single-base extension, staining and scanning (standard Illumina protocols)	9422491020_R03C01_Grn.idat & 9422491020_R03C01_Red.idat	GSM4056725	CLL_C_del17p_refractory_5	Raw data filtering, background subtraction (Noob) and normalization (BMIQ)	Data matrix of beta-values
C (del(17p), refractory)	10PB2975	NK	Male	58	Yes	Refractory	95.71	Yes	10.0	No	0.5	No	2.5	Yes	53.50	Yes	39.00	c.159_185del27bp; c.833C>G	in frame deletion; missense	2; 45	Isolation of peripheral blood mononuclear cells (PBMCs) from blood samples via density gradient centrifugation (Pancoll human)	Purification of CD19-positive cell fraction using CD19 MicroBeads	Genomic DNA extraction (Qiagen AllPrep kit)	Bisulfite DNA conversion, whole-genome amplification, fragmentation and hybridization to Illumina Infinium HumanMethylation450 BeadChips (standard Illumina protocols)	Single-base extension, staining and scanning (standard Illumina protocols)	9422491041_R05C01_Grn.idat & 9422491041_R05C01_Red.idat	GSM4056753	CLL_C_del17p_refractory_8	Raw data filtering, background subtraction (Noob) and normalization (BMIQ)	Data matrix of beta-values
D (no del(17p), chemosensitive)	08PB0411	Female	Female	62	No	NA	98.2	No	4.0	No	0.0	No	12.0	No	2.00	No	4.00	none detected	NA	0	Isolation of peripheral blood mononuclear cells (PBMCs) from blood samples via density gradient centrifugation (Pancoll human)	Purification of CD19-positive cell fraction using CD19 MicroBeads	Genomic DNA extraction (Qiagen AllPrep kit)	Bisulfite DNA conversion, whole-genome amplification, fragmentation and hybridization to Illumina Infinium HumanMethylation450 BeadChips (standard Illumina protocols)	Single-base extension, staining and scanning (standard Illumina protocols)	9422491005_R05C01_Grn.idat & 9422491005_R05C01_Red.idat	GSM4056705	CLL_D_nodel17p_chemosensitive_2	Raw data filtering, background subtraction (Noob) and normalization (BMIQ)	Data matrix of beta-values
D (no del(17p), chemosensitive)	10PB3262	Male	Male	56	No	NA	100	Yes	26.0	No	1.0	No	1.0	No	1.00	No	5.50	none detected	NA	0	Isolation of peripheral blood mononuclear cells (PBMCs) from blood samples via density gradient centrifugation (Pancoll human)	Purification of CD19-positive cell fraction using CD19 MicroBeads	Genomic DNA extraction (Qiagen AllPrep kit)	Bisulfite DNA conversion, whole-genome amplification, fragmentation and hybridization to Illumina Infinium HumanMethylation450 BeadChips (standard Illumina protocols)	Single-base extension, staining and scanning (standard Illumina protocols)	9422491020_R05C01_Grn.idat & 9422491020_R05C01_Red.idat	GSM4056729	CLL_D_nodel17p_chemosensitive_9	Raw data filtering, background subtraction (Noob) and normalization (BMIQ)	Data matrix of beta-values
D (no del(17p), chemosensitive)	08PB1082	Male	Male	66	No	NA	99.53	Yes	87.5	No	1.0	No	2.5	No	11.00	No	4.00	none detected	NA	0	Isolation of peripheral blood mononuclear cells (PBMCs) from blood samples via density gradient centrifugation (Pancoll human)	Purification of CD19-positive cell fraction using CD19 MicroBeads	Genomic DNA extraction (Qiagen AllPrep kit)	Bisulfite DNA conversion, whole-genome amplification, fragmentation and hybridization to Illumina Infinium HumanMethylation450 BeadChips (standard Illumina protocols)	Single-base extension, staining and scanning (standard Illumina protocols)	9422491041_R01C02_Grn.idat & 9422491041_R01C02_Red.idat	GSM4056746	CLL_D_nodel17p_chemosensitive_14	Raw data filtering, background subtraction (Noob) and normalization (BMIQ)	Data matrix of beta-values
D (no del(17p), chemosensitive)	09PB0626	Male	Male	59	Yes	Responded	100	No	6.5	No	1.5	No	2.5	No	0.50	No	1.50	none detected	NA	0	Isolation of peripheral blood mononuclear cells (PBMCs) from blood samples via density gradient centrifugation (Pancoll human)	Purification of CD19-positive cell fraction using CD19 MicroBeads	Genomic DNA extraction (Qiagen AllPrep kit)	Bisulfite DNA conversion, whole-genome amplification, fragmentation and hybridization to Illumina Infinium HumanMethylation450 BeadChips (standard Illumina protocols)	Single-base extension, staining and scanning (standard Illumina protocols)	9422491005_R04C02_Grn.idat & 9422491005_R04C02_Red.idat	GSM4056704	CLL_D_nodel17p_chemosensitive_1	Raw data filtering, background subtraction (Noob) and normalization (BMIQ)	Data matrix of beta-values
D (no del(17p), chemosensitive)	09PB0700	Male	Male	65	No	NA	100	Yes	79.0	No	1.5	No	7.0	No	1.50	No	2.50	none detected	NA	0	Isolation of peripheral blood mononuclear cells (PBMCs) from blood samples via density gradient centrifugation (Pancoll human)	Purification of CD19-positive cell fraction using CD19 MicroBeads	Genomic DNA extraction (Qiagen AllPrep kit)	Bisulfite DNA conversion, whole-genome amplification, fragmentation and hybridization to Illumina Infinium HumanMethylation450 BeadChips (standard Illumina protocols)	Single-base extension, staining and scanning (standard Illumina protocols)	9422491020_R04C02_Grn.idat & 9422491020_R04C02_Red.idat	GSM4056728	CLL_D_nodel17p_chemosensitive_8	Raw data filtering, background subtraction (Noob) and normalization (BMIQ)	Data matrix of beta-values
D (no del(17p), chemosensitive)	08PB2670	Female	Female	73	No	NA	94.06	Yes	56.0	No	1.0	No	1.0	No	0.50	No	4.00	none detected	NA	0	Isolation of peripheral blood mononuclear cells (PBMCs) from blood samples via density gradient centrifugation (Pancoll human)	Purification of CD19-positive cell fraction using CD19 MicroBeads	Genomic DNA extraction (Qiagen AllPrep kit)	Bisulfite DNA conversion, whole-genome amplification, fragmentation and hybridization to Illumina Infinium HumanMethylation450 BeadChips (standard Illumina protocols)	Single-base extension, staining and scanning (standard Illumina protocols)	9422491041_R04C02_Grn.idat & 9422491041_R04C02_Red.idat	GSM4056752	CLL_D_nodel17p_chemosensitive_16	Raw data filtering, background subtraction (Noob) and normalization (BMIQ)	Data matrix of beta-values
D (no del(17p), chemosensitive)	09PB1646	Male	Male	52	No	NA	100	No	1.5	No	1.0	No	5.0	No	2.00	No	4.00	none detected	NA	0	Isolation of peripheral blood mononuclear cells (PBMCs) from blood samples via density gradient centrifugation (Pancoll human)	Purification of CD19-positive cell fraction using CD19 MicroBeads	Genomic DNA extraction (Qiagen AllPrep kit)	Bisulfite DNA conversion, whole-genome amplification, fragmentation and hybridization to Illumina Infinium HumanMethylation450 BeadChips (standard Illumina protocols)	Single-base extension, staining and scanning (standard Illumina protocols)	9422491005_R06C02_Grn.idat & 9422491005_R06C02_Red.idat	GSM4056708	CLL_D_nodel17p_chemosensitive_3	Raw data filtering, background subtraction (Noob) and normalization (BMIQ)	Data matrix of beta-values
D (no del(17p), chemosensitive)	08PB3739	Male	Male	38	No	NA	93.81	No	5.5	No	4.0	No	4.0	No	4.00	No	4.50	none detected	NA	0	Isolation of peripheral blood mononuclear cells (PBMCs) from blood samples via density gradient centrifugation (Pancoll human)	Purification of CD19-positive cell fraction using CD19 MicroBeads	Genomic DNA extraction (Qiagen AllPrep kit)	Bisulfite DNA conversion, whole-genome amplification, fragmentation and hybridization to Illumina Infinium HumanMethylation450 BeadChips (standard Illumina protocols)	Single-base extension, staining and scanning (standard Illumina protocols)	9422491020_R06C02_Grn.idat & 9422491020_R06C02_Red.idat	GSM4056732	CLL_D_nodel17p_chemosensitive_10	Raw data filtering, background subtraction (Noob) and normalization (BMIQ)	Data matrix of beta-values
D (no del(17p), chemosensitive)	09PB1757	Male	Male	72	Yes	Responded	100	No	4.0	No	2.5	No	2.5	No	1.50	No	2.00	none detected	NA	0	Isolation of peripheral blood mononuclear cells (PBMCs) from blood samples via density gradient centrifugation (Pancoll human)	Purification of CD19-positive cell fraction using CD19 MicroBeads	Genomic DNA extraction (Qiagen AllPrep kit)	Bisulfite DNA conversion, whole-genome amplification, fragmentation and hybridization to Illumina Infinium HumanMethylation450 BeadChips (standard Illumina protocols)	Single-base extension, staining and scanning (standard Illumina protocols)	9422491041_R06C02_Grn.idat & 9422491041_R06C02_Red.idat	GSM4056756	CLL_D_nodel17p_chemosensitive_17	Raw data filtering, background subtraction (Noob) and normalization (BMIQ)	Data matrix of beta-values
D (no del(17p), chemosensitive)	08PB4270	Female	Female	43	No	NA	94.59	Yes	20.0	No	2.5	No	4.5	No	1.50	No	5.50	none detected	NA	0	Isolation of peripheral blood mononuclear cells (PBMCs) from blood samples via density gradient centrifugation (Pancoll human)	Purification of CD19-positive cell fraction using CD19 MicroBeads	Genomic DNA extraction (Qiagen AllPrep kit)	Bisulfite DNA conversion, whole-genome amplification, fragmentation and hybridization to Illumina Infinium HumanMethylation450 BeadChips (standard Illumina protocols)	Single-base extension, staining and scanning (standard Illumina protocols)	9422491007_R02C01_Grn.idat & 9422491007_R02C01_Red.idat	GSM4056711	CLL_D_nodel17p_chemosensitive_5	Raw data filtering, background subtraction (Noob) and normalization (BMIQ)	Data matrix of beta-values
D (no del(17p), chemosensitive)	08PB5124	Female	Male	84	No	NA	100	No	3.0	No	3.5	No	4.5	No	3.50	No	3.00	none detected	NA	0	Isolation of peripheral blood mononuclear cells (PBMCs) from blood samples via density gradient centrifugation (Pancoll human)	Purification of CD19-positive cell fraction using CD19 MicroBeads	Genomic DNA extraction (Qiagen AllPrep kit)	Bisulfite DNA conversion, whole-genome amplification, fragmentation and hybridization to Illumina Infinium HumanMethylation450 BeadChips (standard Illumina protocols)	Single-base extension, staining and scanning (standard Illumina protocols)	9422491034_R05C01_Grn.idat & 9422491034_R05C01_Red.idat	GSM4056741	CLL_D_nodel17p_chemosensitive_12	Raw data filtering, background subtraction (Noob) and normalization (BMIQ)	Data matrix of beta-values
D (no del(17p), chemosensitive)	08PB7544	Male	Male	41	No	NA	100	Yes	19.0	No	0.0	No	3.0	No	4.50	No	5.50	none detected	NA	0	Isolation of peripheral blood mononuclear cells (PBMCs) from blood samples via density gradient centrifugation (Pancoll human)	Purification of CD19-positive cell fraction using CD19 MicroBeads	Genomic DNA extraction (Qiagen AllPrep kit)	Bisulfite DNA conversion, whole-genome amplification, fragmentation and hybridization to Illumina Infinium HumanMethylation450 BeadChips (standard Illumina protocols)	Single-base extension, staining and scanning (standard Illumina protocols)	9422491055_R05C01_Grn.idat & 9422491055_R05C01_Red.idat	GSM4056765	CLL_D_nodel17p_chemosensitive_19	Raw data filtering, background subtraction (Noob) and normalization (BMIQ)	Data matrix of beta-values
D (no del(17p), chemosensitive)	09PB7511	Male	Male	71	No	NA	87.39	Yes	83.0	No	0.0	No	0.0	No	2.00	No	3.50	none detected	NA	0	Isolation of peripheral blood mononuclear cells (PBMCs) from blood samples via density gradient centrifugation (Pancoll human)	Purification of CD19-positive cell fraction using CD19 MicroBeads	Genomic DNA extraction (Qiagen AllPrep kit)	Bisulfite DNA conversion, whole-genome amplification, fragmentation and hybridization to Illumina Infinium HumanMethylation450 BeadChips (standard Illumina protocols)	Single-base extension, staining and scanning (standard Illumina protocols)	9422491007_R01C02_Grn.idat & 9422491007_R01C02_Red.idat	GSM4056710	CLL_D_nodel17p_chemosensitive_4	Raw data filtering, background subtraction (Noob) and normalization (BMIQ)	Data matrix of beta-values
D (no del(17p), chemosensitive)	08PB8031	Female	Female	63	No	NA	81.48	No	5.0	No	0.0	No	2.5	No	3.00	No	3.00	none detected	NA	0	Isolation of peripheral blood mononuclear cells (PBMCs) from blood samples via density gradient centrifugation (Pancoll human)	Purification of CD19-positive cell fraction using CD19 MicroBeads	Genomic DNA extraction (Qiagen AllPrep kit)	Bisulfite DNA conversion, whole-genome amplification, fragmentation and hybridization to Illumina Infinium HumanMethylation450 BeadChips (standard Illumina protocols)	Single-base extension, staining and scanning (standard Illumina protocols)	9422491034_R04C02_Grn.idat & 9422491034_R04C02_Red.idat	GSM4056740	CLL_D_nodel17p_chemosensitive_11	Raw data filtering, background subtraction (Noob) and normalization (BMIQ)	Data matrix of beta-values
D (no del(17p), chemosensitive)	08PB3517	Male	Male	68	Yes	Responded	99.55	Yes	82.0	No	4.0	No	0.0	No	5.00	No	3.50	none detected	NA	0	Isolation of peripheral blood mononuclear cells (PBMCs) from blood samples via density gradient centrifugation (Pancoll human)	Purification of CD19-positive cell fraction using CD19 MicroBeads	Genomic DNA extraction (Qiagen AllPrep kit)	Bisulfite DNA conversion, whole-genome amplification, fragmentation and hybridization to Illumina Infinium HumanMethylation450 BeadChips (standard Illumina protocols)	Single-base extension, staining and scanning (standard Illumina protocols)	9422491055_R01C02_Grn.idat & 9422491055_R01C02_Red.idat	GSM4056758	CLL_D_nodel17p_chemosensitive_18	Raw data filtering, background subtraction (Noob) and normalization (BMIQ)	Data matrix of beta-values
D (no del(17p), chemosensitive)	12PB0638	Male	Male	51	Yes	Responded	100	Yes	9.5	No	0.5	No	1.5	No	2.00	No	3.50	none detected	NA	0	Isolation of peripheral blood mononuclear cells (PBMCs) from blood samples via density gradient centrifugation (Pancoll human)	Purification of CD19-positive cell fraction using CD19 MicroBeads	Genomic DNA extraction (Qiagen AllPrep kit)	Bisulfite DNA conversion, whole-genome amplification, fragmentation and hybridization to Illumina Infinium HumanMethylation450 BeadChips (standard Illumina protocols)	Single-base extension, staining and scanning (standard Illumina protocols)	9422491007_R04C02_Grn.idat & 9422491007_R04C02_Red.idat	GSM4056716	CLL_D_nodel17p_chemosensitive_6	Raw data filtering, background subtraction (Noob) and normalization (BMIQ)	Data matrix of beta-values
D (no del(17p), chemosensitive)	07PB1887*	Male	Male	58	Yes	Responded	100	No	6.0	No	1.0	No	3.0	No	0.50	No	4.00	none detected	NA	0	Isolation of peripheral blood mononuclear cells (PBMCs) from blood samples via density gradient centrifugation (Pancoll human)	Purification of CD19-positive cell fraction using CD19 MicroBeads	Genomic DNA extraction (Qiagen AllPrep kit)	Bisulfite DNA conversion, whole-genome amplification, fragmentation and hybridization to Illumina Infinium HumanMethylation450 BeadChips (standard Illumina protocols)	Single-base extension, staining and scanning (standard Illumina protocols)	9422491034_R06C02_Grn.idat & 9422491034_R06C02_Red.idat	GSM4056744	CLL_D_nodel17p_chemosensitive_13_timepoint1	Raw data filtering, background subtraction (Noob) and normalization (BMIQ)	Data matrix of beta-values
D (no del(17p), chemosensitive)	10PB6041*	Male	Male	61	Yes	Responded	100	Yes	16.0	No	2.5	No	1.5	No	5.50	No	5.00	none detected	NA	0	Isolation of peripheral blood mononuclear cells (PBMCs) from blood samples via density gradient centrifugation (Pancoll human)	Purification of CD19-positive cell fraction using CD19 MicroBeads	Genomic DNA extraction (Qiagen AllPrep kit)	Bisulfite DNA conversion, whole-genome amplification, fragmentation and hybridization to Illumina Infinium HumanMethylation450 BeadChips (standard Illumina protocols)	Single-base extension, staining and scanning (standard Illumina protocols)	9422491055_R03C02_Grn.idat & 9422491055_R03C02_Red.idat	GSM4056762	CLL_D_nodel17p_chemosensitive_13_timepoint2	Raw data filtering, background subtraction (Noob) and normalization (BMIQ)	Data matrix of beta-values
D (no del(17p), chemosensitive)	12PB8783	Female	Female	62	No	NA	100	No	4.5	No	2.0	No	0.0	No	4.00	No	7.50	none detected	NA	0	Isolation of peripheral blood mononuclear cells (PBMCs) from blood samples via density gradient centrifugation (Pancoll human)	Purification of CD19-positive cell fraction using CD19 MicroBeads	Genomic DNA extraction (Qiagen AllPrep kit)	Bisulfite DNA conversion, whole-genome amplification, fragmentation and hybridization to Illumina Infinium HumanMethylation450 BeadChips (standard Illumina protocols)	Single-base extension, staining and scanning (standard Illumina protocols)	9422491007_R06C02_Grn.idat & 9422491007_R06C02_Red.idat	GSM4056720	CLL_D_nodel17p_chemosensitive_7	Raw data filtering, background subtraction (Noob) and normalization (BMIQ)	Data matrix of beta-values
D (no del(17p), chemosensitive)	13PB11267	Male	Male	64	Yes	Responded	100	No	4.5	No	0.0	No	8.0	No	5.00	No	4.00	none detected	NA	0	Isolation of peripheral blood mononuclear cells (PBMCs) from blood samples via density gradient centrifugation (Pancoll human)	Purification of CD19-positive cell fraction using CD19 MicroBeads	Genomic DNA extraction (Qiagen AllPrep kit)	Bisulfite DNA conversion, whole-genome amplification, fragmentation and hybridization to Illumina Infinium HumanMethylation450 BeadChips (standard Illumina protocols)	Single-base extension, staining and scanning (standard Illumina protocols)	9422491041_R02C01_Grn.idat & 9422491041_R02C01_Red.idat	GSM4056747	CLL_D_nodel17p_chemosensitive_15	Raw data filtering, background subtraction (Noob) and normalization (BMIQ)	Data matrix of beta-values
D (no del(17p), chemosensitive)	12PB7846	Male	Male	45	No	NA	100	Yes	67.0	No	1.0	No	0.5	No	0.00	No	3.50	none detected	NA	0	Isolation of peripheral blood mononuclear cells (PBMCs) from blood samples via density gradient centrifugation (Pancoll human)	Purification of CD19-positive cell fraction using CD19 MicroBeads	Genomic DNA extraction (Qiagen AllPrep kit)	Bisulfite DNA conversion, whole-genome amplification, fragmentation and hybridization to Illumina Infinium HumanMethylation450 BeadChips (standard Illumina protocols)	Single-base extension, staining and scanning (standard Illumina protocols)	9422491055_R06C02_Grn.idat & 9422491055_R06C02_Red.idat	GSM4056768	CLL_D_nodel17p_chemosensitive_20	Raw data filtering, background subtraction (Noob) and normalization (BMIQ)	Data matrix of beta-values
E (no del(17p), refractory)	08PB4647	Male	Male	60	Yes	Refractory	97.7	Yes	91.0	No	2.5	No	1.0	No	0.50	No	2.50	none detected	NA	0	Isolation of peripheral blood mononuclear cells (PBMCs) from blood samples via density gradient centrifugation (Pancoll human)	Purification of CD19-positive cell fraction using CD19 MicroBeads	Genomic DNA extraction (Qiagen AllPrep kit)	Bisulfite DNA conversion, whole-genome amplification, fragmentation and hybridization to Illumina Infinium HumanMethylation450 BeadChips (standard Illumina protocols)	Single-base extension, staining and scanning (standard Illumina protocols)	9422491005_R04C01_Grn.idat & 9422491005_R04C01_Red.idat	GSM4056703	CLL_E_nodel17p_refractory_2	Raw data filtering, background subtraction (Noob) and normalization (BMIQ)	Data matrix of beta-values
E (no del(17p), refractory)	08PB8276	Male	Male	66	Yes	Refractory	99.57	Yes	96.0	No	1.0	No	1.5	No	2.50	No	2.50	none detected	NA	0	Isolation of peripheral blood mononuclear cells (PBMCs) from blood samples via density gradient centrifugation (Pancoll human)	Purification of CD19-positive cell fraction using CD19 MicroBeads	Genomic DNA extraction (Qiagen AllPrep kit)	Bisulfite DNA conversion, whole-genome amplification, fragmentation and hybridization to Illumina Infinium HumanMethylation450 BeadChips (standard Illumina protocols)	Single-base extension, staining and scanning (standard Illumina protocols)	9422491020_R04C01_Grn.idat & 9422491020_R04C01_Red.idat	GSM4056727	CLL_E_nodel17p_refractory_7	Raw data filtering, background subtraction (Noob) and normalization (BMIQ)	Data matrix of beta-values
E (no del(17p), refractory)	09PB7242	NK	Male	46	Yes	Refractory	99.53	No	4.0	No	2.5	No	6.5	No	1.50	No	5.00	none detected	NA	0	Isolation of peripheral blood mononuclear cells (PBMCs) from blood samples via density gradient centrifugation (Pancoll human)	Purification of CD19-positive cell fraction using CD19 MicroBeads	Genomic DNA extraction (Qiagen AllPrep kit)	Bisulfite DNA conversion, whole-genome amplification, fragmentation and hybridization to Illumina Infinium HumanMethylation450 BeadChips (standard Illumina protocols)	Single-base extension, staining and scanning (standard Illumina protocols)	9422491041_R01C01_Grn.idat & 9422491041_R01C01_Red.idat	GSM4056745	CLL_E_nodel17p_refractory_11	Raw data filtering, background subtraction (Noob) and normalization (BMIQ)	Data matrix of beta-values
E (no del(17p), refractory)	09PB0906	Male	Male	68	Yes	Refractory	98.98	Yes	38.0	No	5.0	No	2.5	Yes	94.00	No	4.00	none detected	NA	0	Isolation of peripheral blood mononuclear cells (PBMCs) from blood samples via density gradient centrifugation (Pancoll human)	Purification of CD19-positive cell fraction using CD19 MicroBeads	Genomic DNA extraction (Qiagen AllPrep kit)	Bisulfite DNA conversion, whole-genome amplification, fragmentation and hybridization to Illumina Infinium HumanMethylation450 BeadChips (standard Illumina protocols)	Single-base extension, staining and scanning (standard Illumina protocols)	9422491005_R03C02_Grn.idat & 9422491005_R03C02_Red.idat	GSM4056702	CLL_E_nodel17p_refractory_1	Raw data filtering, background subtraction (Noob) and normalization (BMIQ)	Data matrix of beta-values
E (no del(17p), refractory)	11PB2066	NK	Male	71	Yes	Refractory	98.3	Yes	80.5	No	1.5	No	0.5	No	2.00	No	4.50	none detected	NA	0	Isolation of peripheral blood mononuclear cells (PBMCs) from blood samples via density gradient centrifugation (Pancoll human)	Purification of CD19-positive cell fraction using CD19 MicroBeads	Genomic DNA extraction (Qiagen AllPrep kit)	Bisulfite DNA conversion, whole-genome amplification, fragmentation and hybridization to Illumina Infinium HumanMethylation450 BeadChips (standard Illumina protocols)	Single-base extension, staining and scanning (standard Illumina protocols)	9422491020_R03C02_Grn.idat & 9422491020_R03C02_Red.idat	GSM4056726	CLL_E_nodel17p_refractory_6	Raw data filtering, background subtraction (Noob) and normalization (BMIQ)	Data matrix of beta-values
E (no del(17p), refractory)	11PB11934	NK	Female	70	Yes	Refractory	100	Yes	31.5	No	1.0	No	0.0	Yes	96.50	No	4.00	none detected	NA	0	Isolation of peripheral blood mononuclear cells (PBMCs) from blood samples via density gradient centrifugation (Pancoll human)	Purification of CD19-positive cell fraction using CD19 MicroBeads	Genomic DNA extraction (Qiagen AllPrep kit)	Bisulfite DNA conversion, whole-genome amplification, fragmentation and hybridization to Illumina Infinium HumanMethylation450 BeadChips (standard Illumina protocols)	Single-base extension, staining and scanning (standard Illumina protocols)	9422491041_R06C01_Grn.idat & 9422491041_R06C01_Red.idat	GSM4056755	CLL_E_nodel17p_refractory_13	Raw data filtering, background subtraction (Noob) and normalization (BMIQ)	Data matrix of beta-values
E (no del(17p), refractory)	10PB6173	NK	Female	57	Yes	Refractory	99.54	Yes	14.0	No	2.5	No	2.5	No	1.00	No	4.00	none detected	NA	0	Isolation of peripheral blood mononuclear cells (PBMCs) from blood samples via density gradient centrifugation (Pancoll human)	Purification of CD19-positive cell fraction using CD19 MicroBeads	Genomic DNA extraction (Qiagen AllPrep kit)	Bisulfite DNA conversion, whole-genome amplification, fragmentation and hybridization to Illumina Infinium HumanMethylation450 BeadChips (standard Illumina protocols)	Single-base extension, staining and scanning (standard Illumina protocols)	9422491007_R01C01_Grn.idat & 9422491007_R01C01_Red.idat	GSM4056709	CLL_E_nodel17p_refractory_3	Raw data filtering, background subtraction (Noob) and normalization (BMIQ)	Data matrix of beta-values
E (no del(17p), refractory)	10PB10381	Male	Male	46	Yes	Refractory	100	Yes	76.5	No	1.0	No	3.0	Yes	60.00	No	3.00	none detected	NA	0	Isolation of peripheral blood mononuclear cells (PBMCs) from blood samples via density gradient centrifugation (Pancoll human)	Genomic DNA extraction (Qiagen AllPrep kit)	Bisulfite DNA conversion, whole-genome amplification, fragmentation and hybridization to Illumina Infinium HumanMethylation450 BeadChips (standard Illumina protocols)	Single-base extension, staining and scanning (standard Illumina protocols)	9422491020_R05C02_Grn.idat & 9422491020_R05C02_Red.idat	GSM4056730	CLL_E_nodel17p_refractory_8	Raw data filtering, background subtraction (Noob) and normalization (BMIQ)	Data matrix of beta-values	
E (no del(17p), refractory)	10PB1329	NK	Male	52	Yes	Refractory	100	Yes	13.5	No	2.5	Yes	55.5	No	7.50	No	5.00	none detected	NA	0	Isolation of peripheral blood mononuclear cells (PBMCs) from blood samples via density gradient centrifugation (Pancoll human)	Genomic DNA extraction (Qiagen AllPrep kit)	Bisulfite DNA conversion, whole-genome amplification, fragmentation and hybridization to Illumina Infinium HumanMethylation450 BeadChips (standard Illumina protocols)	Single-base extension, staining and scanning (standard Illumina protocols)	9422491041_R03C02_Grn.idat & 9422491041_R03C02_Red.idat	GSM4056750	CLL_E_nodel17p_refractory_12	Raw data filtering, background subtraction (Noob) and normalization (BMIQ)	Data matrix of beta-values	
E (no del(17p), refractory)	10PB2885	NK	Female	65	Yes	Refractory	96.13	Yes	84.5	No	4.5	No	2.0	No	4.00	No	6.00	none detected	NA	0	Isolation of peripheral blood mononuclear cells (PBMCs) from blood samples via density gradient centrifugation (Pancoll human)	Purification of CD19-positive cell fraction using CD19 MicroBeads	Genomic DNA extraction (Qiagen AllPrep kit)	Bisulfite DNA conversion, whole-genome amplification, fragmentation and hybridization to Illumina Infinium HumanMethylation450 BeadChips (standard Illumina protocols)	Single-base extension, staining and scanning (standard Illumina protocols)	9422491007_R06C01_Grn.idat & 9422491007_R06C01_Red.idat	GSM4056719	CLL_E_nodel17p_refractory_5	Raw data filtering, background subtraction (Noob) and normalization (BMIQ)	Data matrix of beta-values
E (no del(17p), refractory)	08PB6113	Female	Female	38	Yes	Refractory	100	No	3.5	No	1.0	No	0.0	No	4.50	No	7.00	none detected	NA	0	Isolation of peripheral blood mononuclear cells (PBMCs) from blood samples via density gradient centrifugation (Pancoll human)	Genomic DNA extraction (Qiagen AllPrep kit)	Bisulfite DNA conversion, whole-genome amplification, fragmentation and hybridization to Illumina Infinium HumanMethylation450 BeadChips (standard Illumina protocols)	Single-base extension, staining and scanning (standard Illumina protocols)	9422491034_R04C01_Grn.idat & 9422491034_R04C01_Red.idat	GSM4056739	CLL_E_nodel17p_refractory_10	Raw data filtering, background subtraction (Noob) and normalization (BMIQ)	Data matrix of beta-values	
E (no del(17p), refractory)	08PB5904	Male	Male	69	Yes	Refractory	99.06	Yes	87.0	No	1.5	No	0.5	Yes	89.50	No	4.00	none detected	NA	0	Isolation of peripheral blood mononuclear cells (PBMCs) from blood samples via density gradient centrifugation (Pancoll human)	Purification of CD19-positive cell fraction using CD19 MicroBeads	Genomic DNA extraction (Qiagen AllPrep kit)	Bisulfite DNA conversion, whole-genome amplification, fragmentation and hybridization to Illumina Infinium HumanMethylation450 BeadChips (standard Illumina protocols)	Single-base extension, staining and scanning (standard Illumina protocols)	9422491055_R04C01_Grn.idat & 9422491055_R04C01_Red.idat	GSM4056763	CLL_E_nodel17p_refractory_14	Raw data filtering, background subtraction (Noob) and normalization (BMIQ)	Data matrix of beta-values
E (no del(17p), refractory)	11PB13477	Male	Male	66	Yes	Refractory	100	Yes	91.5	No	0.0	No	1.5	No	3.00	No	3.00	none detected	NA	0	Isolation of peripheral blood mononuclear cells (PBMCs) from blood samples via density gradient centrifugation (Pancoll human)	Purification of CD19-positive cell fraction using CD19 MicroBeads	Genomic DNA extraction (Qiagen AllPrep kit)	Bisulfite DNA conversion, whole-genome amplification, fragmentation and hybridization to Illumina Infinium HumanMethylation450 BeadChips (standard Illumina protocols)	Single-base extension, staining and scanning (standard Illumina protocols)	9422491007_R03C02_Grn.idat & 9422491007_R03C02_Red.idat	GSM4056714	CLL_E_nodel17p_refractory_4	Raw data filtering, background subtraction (Noob) and normalization (BMIQ)	Data matrix of beta-values
E (no del(17p), refractory)	07PB2880	Male	Male	63	Yes	Refractory	95.95	Yes	97.0	No	0.0	No	0.5	No	3.00	No	2.50	none detected	NA	0	Isolation of peripheral blood mononuclear cells (PBMCs) from blood samples via density gradient centrifugation (Pancoll human)	Purification of CD19-positive cell fraction using CD19 MicroBeads	Genomic DNA extraction (Qiagen AllPrep kit)	Bisulfite DNA conversion, whole-genome amplification, fragmentation and hybridization to Illumina Infinium HumanMethylation450 BeadChips (standard Illumina protocols)	Single-base extension, staining and scanning (standard Illumina protocols)	9422491034_R03C02_Grn.idat & 9422491034_R03C02_Red.idat	GSM4056738	CLL_E_nodel17p_refractory_9	Raw data filtering, background subtraction (Noob) and normalization (BMIQ)	Data matrix of beta-values
E (no del(17p), refractory)	07PB6512	Male	Male	59	Yes	Refractory	100	Yes	44.0	No	5.0	No	7.0	No	7.50	No	5.00	none detected	NA	0	Isolation of peripheral blood mononuclear cells (PBMCs) from blood samples via density gradient centrifugation (Pancoll human)	Purification of CD19-positive cell fraction using CD19 MicroBeads	Genomic DNA extraction (Qiagen AllPrep kit)	Bisulfite DNA conversion, whole-genome amplification, fragmentation and hybridization to Illumina Infinium HumanMethylation450 BeadChips (standard Illumina protocols)	Single-base extension, staining and scanning (standard Illumina protocols)	9422491055_R05C02_Grn.idat & 9422491055_R05C02_Red.idat	GSM4056766	CLL_E_nodel17p_refractory_15	Raw data filtering, background subtraction (Noob) and normalization (BMIQ)	Data matrix of beta-values

NA = not applicable, NK = Not known.

*Samples 07PB1887 and 10PB6041 were taken from the same patient with a time difference of 40 months. There was cytogenetic progression from normal karyotype to del13q14. Only the first sample (07PB1887) is included in the summary characteristics provided in Table 1.

^Gender was not available for a part of the anonymized patients' samples.

We used the gender prediction algorithm in RnBeads to predict patients' sex.

**Online-only Table 2:** DNA concentrations and quality control parameters measured using a NanoDrop ND-1000 UV-Vis Spectrophotometer. Each sample was measured in triplicates.

Sample ID	ng/µl	A260	A280	260/280	260/230	Constant	Cursor Pos.	Cursor abs.	340 raw
09PB8587	237.8	4.756	2.472	1.92	2.04	50	230	2.328	−0.006
09PB8587	242.24	4.845	2.531	1.91	2.01	50	230	2.414	0.02
09PB8587	243.92	4.878	2.53	1.93	2.05	50	230	2.383	0.006
11PB5152	94.52	1.89	0.997	1.9	1.98	50	230	0.957	−0.01
11PB5152	93.13	1.863	1.015	1.84	2.05	50	230	0.908	0.001
11PB5152	87.66	1.753	0.942	1.86	1.99	50	230	0.88	−0.022
09PB7427	167.37	3.347	1.74	1.92	2.12	50	230	1.578	0.022
09PB7427	164.15	3.283	1.706	1.92	2.17	50	230	1.511	0.03
09PB7427	162.06	3.241	1.654	1.96	2.16	50	230	1.501	0.043
08PB4647	141.64	2.833	1.473	1.92	2	50	230	1.415	0.044
08PB4647	137.1	2.742	1.432	1.91	2	50	230	1.368	0.02
08PB4647	137.33	2.747	1.418	1.94	2.03	50	230	1.356	0.018
10PB5506	169.28	3.386	1.781	1.9	2.12	50	230	1.598	0.015
10PB5506	158.03	3.161	1.632	1.94	2.17	50	230	1.455	0.007
10PB5506	154.57	3.091	1.615	1.91	2.09	50	230	1.48	0.012
09PB6581	170.2	3.404	1.776	1.92	2.07	50	230	1.644	0.012
09PB6581	177.76	3.555	1.866	1.91	2.11	50	230	1.683	−0.001
09PB6581	168.37	3.367	1.763	1.91	2.1	50	230	1.606	0.007
11PB1528	297.15	5.943	3.105	1.91	2.06	50	230	2.887	−0.054
11PB1528	296.77	5.935	3.088	1.92	2.07	50	230	2.871	−0.052
11PB1528	266.53	5.331	2.795	1.91	2.03	50	230	2.625	−0.057
08PB8276	663.84	13.277	7.061	1.88	2.23	50	230	5.941	0.206
08PB8276	652.39	13.048	6.983	1.87	2.21	50	230	5.906	0.171
08PB8276	675.04	13.501	7.219	1.87	2.21	50	230	6.108	0.151
10PB8858	169.94	3.399	1.786	1.9	2.01	50	230	1.687	−0.033
10PB8858	166.87	3.337	1.746	1.91	2.08	50	230	1.606	−0.029
10PB8858	165.86	3.317	1.712	1.94	2.1	50	230	1.576	−0.022
09PB2750	59.26	1.185	0.635	1.87	0.68	50	230	1.736	−0.037
09PB2750	58.97	1.179	0.636	1.85	0.7	50	230	1.68	−0.032
09PB2750	60.67	1.213	0.613	1.98	0.72	50	230	1.693	−0.034
08PB0411	129.81	2.596	1.35	1.92	2.04	50	230	1.272	0.003
08PB0411	124.6	2.492	1.298	1.92	1.97	50	230	1.267	0.09
08PB0411	127.42	2.548	1.337	1.91	1.99	50	230	1.281	0.01
09PB3261	225.27	4.505	2.341	1.92	1.69	50	230	2.661	−0.03
09PB3261	225.73	4.515	2.331	1.94	1.7	50	230	2.655	−0.038
09PB3261	228.23	4.565	2.374	1.92	1.68	50	230	2.722	−0.029
09PB7242	130.02	2.6	1.374	1.89	2.15	50	230	1.208	0.023
09PB7242	124.29	2.486	1.305	1.9	2.16	50	230	1.15	−0.001
09PB7242	145.46	2.909	1.536	1.89	2.21	50	230	1.316	−0.007
08PB1082	124.97	2.499	1.33	1.88	2.03	50	230	1.233	0
08PB1082	123.44	2.469	1.32	1.87	2.01	50	230	1.229	0.026
08PB1082	125.76	2.515	1.336	1.88	2.06	50	230	1.219	−0.009
09PB6227	130.08	2.602	1.366	1.9	2.07	50	230	1.254	0
09PB6227	131.68	2.634	1.386	1.9	2.08	50	230	1.267	−0.008
09PB6227	135.02	2.7	1.442	1.87	2.01	50	230	1.344	−0.02
10PB0115	160.62	3.212	1.713	1.88	2.14	50	230	1.504	−0.018
10PB0115	154.96	3.099	1.663	1.86	2.11	50	230	1.468	−0.009
10PB0115	138.74	2.775	1.452	1.91	2.14	50	230	1.298	−0.018
10PB8700	184.12	3.682	1.915	1.92	2.11	50	230	1.749	−0.007
10PB8700	185.67	3.713	1.932	1.92	2.16	50	230	1.723	−0.013
10PB8700	189.23	3.785	1.976	1.91	2.18	50	230	1.739	−0.022
09PB0906	103.3	2.066	1.101	1.88	1.76	50	230	1.175	−0.022
09PB0906	100.68	2.014	1.069	1.88	1.73	50	230	1.161	−0.029
09PB0906	103.97	2.079	1.114	1.87	1.75	50	230	1.187	−0.014
09PB0626	178.39	3.568	1.88	1.9	2.12	50	230	1.68	−0.006
09PB0626	174.59	3.492	1.806	1.93	2.06	50	230	1.691	−0.016
09PB0626	197.27	3.945	2.076	1.9	2.19	50	230	1.804	−0.008
09PB0858	314.84	6.297	3.299	1.91	2.19	50	230	2.875	−0.014
09PB0858	294.16	5.883	3.107	1.89	2.12	50	230	2.776	−0.012
09PB0858	274.05	5.481	2.909	1.88	2.13	50	230	2.577	−0.011
10PB2807	138.46	2.769	1.437	1.93	2.13	50	230	1.297	−0.002
10PB2807	136.71	2.734	1.427	1.92	2.07	50	230	1.322	0.005
10PB2807	136.26	2.725	1.45	1.88	2.06	50	230	1.324	−0.019
11PB2066	185.75	3.715	1.944	1.91	2.22	50	230	1.675	0.001
11PB2066	151.89	3.038	1.582	1.92	2.13	50	230	1.424	−0.018
11PB2066	152.21	3.044	1.621	1.88	2.08	50	230	1.466	−0.001
09PB0700	171.36	3.427	1.815	1.89	1.91	50	230	1.797	0.015
09PB0700	167.51	3.35	1.755	1.91	2.08	50	230	1.607	−0.002
09PB0700	189.73	3.795	1.991	1.91	2.08	50	230	1.825	−0.015
10PB3589	131.42	2.628	1.371	1.92	1.92	50	230	1.369	0.006
10PB3589	123.4	2.468	1.295	1.91	1.91	50	230	1.292	0.006
10PB3589	122.48	2.45	1.289	1.9	1.88	50	230	1.306	0.003
10PB1144	269.53	5.391	2.876	1.87	2.16	50	230	2.498	0.032
10PB1144	268.84	5.377	2.829	1.9	2.26	50	230	2.379	0.036
10PB1144	267.25	5.345	2.851	1.87	2.2	50	230	2.429	0.028
11PB11934	147.75	2.955	1.543	1.92	1.79	50	230	1.649	0.028
11PB11934	179	3.58	1.89	1.89	1.79	50	230	1.998	0.041
11PB11934	177.37	3.547	1.884	1.88	1.88	50	230	1.886	0.021
09PB1646	150.98	3.02	1.576	1.92	2.05	50	230	1.473	0.025
09PB1646	146.39	2.928	1.538	1.9	2.08	50	230	1.409	0.022
09PB1646	144.76	2.895	1.521	1.9	2.03	50	230	1.429	0.019
10PB0381	136.57	2.731	1.448	1.89	2.08	50	230	1.314	0.024
10PB0381	129.38	2.588	1.36	1.9	1.96	50	230	1.321	0.097
10PB0381	135.79	2.716	1.462	1.86	2.02	50	230	1.347	0.032
10PB5849	123.27	2.465	1.271	1.94	1.88	50	230	1.312	−0.003
10PB5849	117.2	2.344	1.241	1.89	1.88	50	230	1.244	0.026
10PB5849	122.95	2.459	1.304	1.89	1.92	50	230	1.283	0.025
10PB6173	144.45	2.889	1.527	1.89	2.12	50	230	1.362	0.031
10PB6173	134.68	2.694	1.397	1.93	2.05	50	230	1.315	0.036
10PB6173	136.93	2.739	1.438	1.91	2.1	50	230	1.301	−0.004
08PB3739	140.6	2.812	1.455	1.93	1.96	50	230	1.433	0.006
08PB3739	186.3	3.726	1.97	1.89	2.09	50	230	1.784	0.02
08PB3739	170.65	3.413	1.779	1.92	2.03	50	230	1.684	0.024
10PB0918	106.88	2.138	1.129	1.89	1.87	50	230	1.14	0.007
10PB0918	105.58	2.112	1.105	1.91	1.86	50	230	1.133	0.017
10PB0918	108.59	2.172	1.152	1.89	1.87	50	230	1.164	0.014
10PB10381	90.36	1.807	0.945	1.91	1.9	50	230	0.95	0.008
10PB10381	91.38	1.828	0.98	1.86	1.89	50	230	0.968	0.018
10PB10381	93.91	1.878	0.958	1.96	1.95	50	230	0.966	0.963
09PB1757	107.61	2.152	1.135	1.9	1.74	50	230	1.238	0.009
09PB1757	122.51	2.45	1.279	1.92	1.82	50	230	1.35	0.006
09PB1757	118.49	2.37	1.248	1.9	1.83	50	230	1.292	0.026
10PB0488	117.75	2.355	1.213	1.94	2.08	50	230	1.13	0.01
10PB0488	116.92	2.338	1.196	1.96	2.12	50	230	1.101	0.043
10PB0488	117.08	2.342	1.207	1.94	2.09	50	230	1.118	0.005
11PB1958	128.52	2.57	1.317	1.95	2.06	50	230	1.248	−0.184
11PB1958	131.26	2.625	1.362	1.93	2.1	50	230	1.252	0.03
11PB1958	132.73	2.655	1.381	1.92	2.04	50	230	1.304	0.021
09PB2752	120.89	2.418	1.251	1.93	1.94	50	230	1.244	0.238
09PB2752	112.77	2.255	1.177	1.92	1.93	50	230	1.17	0.019
09PB2752	119.5	2.39	1.24	1.93	1.79	50	230	1.334	0.026
10PB1329	135.09	2.702	1.416	1.91	2	50	230	1.348	0.021
10PB1329	133.02	2.66	1.372	1.94	2.08	50	230	1.281	0.017
10PB1329	133.4	2.668	1.378	1.94	2.11	50	230	1.263	0.02
08PB4270	136.03	2.721	1.423	1.91	2.03	50	230	1.34	0.02
08PB4270	133.77	2.675	1.374	1.95	2.09	50	230	1.28	0.03
08PB4270	122.93	2.459	1.269	1.94	1.98	50	230	1.242	0.176
09PB6550	164.06	3.281	1.706	1.92	2.07	50	230	1.582	0.029
09PB6550	162.45	3.249	1.681	1.93	2.11	50	230	1.54	0.034
09PB6550	159.09	3.182	1.647	1.93	2.11	50	230	1.507	0.048
11PB5972	201.88	4.038	2.093	1.93	1.72	50	230	2.349	0.037
11PB5972	204.9	4.098	2.161	1.9	1.72	50	230	2.388	0.041
11PB5972	207.18	4.144	2.139	1.94	1.71	50	230	2.42	0.042
11PB5602	255.45	5.109	2.659	1.92	2.25	50	230	2.269	0.041
11PB5602	250.73	5.015	2.634	1.9	2.22	50	230	2.261	−0.056
11PB5602	244.12	4.882	2.569	1.9	2.11	50	230	2.315	0.053
10PB2885	107.15	2.143	1.142	1.88	1.99	50	230	1.079	0.044
10PB2885	103.55	2.071	1.066	1.94	1.96	50	230	1.058	0.057
10PB2885	104.68	2.094	1.076	1.94	1.96	50	230	1.066	0.051
08PB5124	125.87	2.517	1.295	1.94	1.79	50	230	1.404	0.057
08PB5124	120.39	2.408	1.26	1.91	1.76	50	230	1.364	0.054
08PB5124	121.32	2.426	1.272	1.91	1.68	50	230	1.448	0.051
08PB7544	220.26	4.405	2.313	1.9	1.94	50	230	2.269	0.055
08PB7544	221.37	4.427	2.326	1.9	2.07	50	230	2.137	0.061
08PB7544	201.62	4.032	2.132	1.89	1.97	50	230	2.042	0.061
09PB7511	145.22	2.904	1.494	1.94	2.08	50	230	1.398	0.017
09PB7511	146.83	2.937	1.534	1.91	2.14	50	230	1.373	0.018
09PB7511	149.23	2.985	1.549	1.93	2.09	50	230	1.425	0.041
08PB8771	129.23	2.585	1.316	1.96	2.16	50	230	1.198	0.021
08PB8771	128.1	2.562	1.325	1.93	2.15	50	230	1.193	0.021
08PB8771	146.59	2.932	1.55	1.89	2.04	50	230	1.439	0.085
08PB0722	129.23	2.585	1.353	1.91	2.07	50	230	1.247	0.011
08PB0722	126.97	2.539	1.323	1.92	2.06	50	230	1.232	0.009
08PB0722	121.42	2.428	1.244	1.95	1.99	50	230	1.22	0.016
10PB6266	108.39	2.168	1.112	1.95	1.54	50	230	1.403	0.004
10PB6266	106.26	2.125	1.088	1.95	1.5	50	230	1.417	0.007
10PB6266	112.6	2.252	1.145	1.97	1.6	50	230	1.405	0.019
08PB6113	166.43	3.329	1.737	1.92	2.09	50	230	1.594	−0.026
08PB6113	149.03	2.981	1.554	1.92	1.99	50	230	1.497	0.011
08PB6113	189.34	3.787	1.963	1.93	2.15	50	230	1.762	0.003
08PB8031	137.03	2.741	1.4	1.96	1.99	50	230	1.379	0.002
08PB8031	141.31	2.826	1.453	1.95	1.98	50	230	1.429	0.008
08PB8031	137.14	2.743	1.418	1.93	2.02	50	230	1.358	0.216
09PB5837	515.13	10.303	5.412	1.9	2.22	50	230	4.64	−0.007
09PB5837	562.42	11.248	5.943	1.89	2.22	50	230	5.074	0.004
09PB5837	560.8	11.216	5.987	1.87	2.18	50	230	5.139	−0.004
09PB4164	109.06	2.181	1.147	1.9	2.14	50	230	1.021	−0.007
09PB4164	112.32	2.246	1.154	1.95	2.16	50	230	1.04	−0.002
09PB4164	117.47	2.349	1.216	1.93	2.11	50	230	1.115	−0.002
10PB0279	152.93	3.059	1.604	1.91	2.1	50	230	1.456	0.004
10PB0279	163.34	3.267	1.69	1.93	2.13	50	230	1.532	0.004
10PB0279	144.31	2.886	1.487	1.94	2.12	50	230	1.365	−0.009
08PB5904	171.4	3.428	1.795	1.91	2.09	50	230	1.643	−0.03
08PB5904	181.86	3.637	1.921	1.89	2.09	50	230	1.739	0.004
08PB5904	180.91	3.618	1.875	1.93	2.11	50	230	1.718	−0.002
08PB3517	181.36	3.627	1.89	1.92	2.18	50	230	1.661	−0.002
08PB3517	163.03	3.261	1.682	1.94	2.16	50	230	1.506	0.001
08PB3517	171.92	3.438	1.775	1.94	2.18	50	230	1.579	−0.003
12PB0638	182	3.64	1.892	1.92	2.02	50	230	1.804	−0.012
12PB0638	139.74	2.795	1.445	1.93	1.94	50	230	1.439	−0.004
12PB0638	150.93	3.019	1.571	1.92	1.96	50	230	1.54	0.008
10PB2738	252.44	5.049	2.66	1.9	2.14	50	230	2.356	0.079
10PB2738	243.53	4.871	2.584	1.88	2.05	50	230	2.375	0.021
10PB2738	251.59	5.032	2.645	1.9	2.21	50	230	2.28	0.011
10PB2975	105.21	2.104	1.106	1.9	1.29	50	230	1.637	0.011
10PB2975	91.55	1.831	0.951	1.93	1.9	50	230	0.962	−0.018
10PB2975	97.22	1.944	1.009	1.93	1.98	50	230	0.984	0.035
11PB13477	231.6	4.632	2.419	1.92	2.21	50	230	2.096	0.018
11PB13477	239.65	4.793	2.506	1.91	2.15	50	230	2.234	0.011
11PB13477	226.53	4.531	2.348	1.93	2.22	50	230	2.044	0.064
07PB1887	198.82	3.976	2.109	1.89	2.12	50	230	1.88	0.013
07PB1887	187.69	3.754	1.959	1.92	2.16	50	230	1.739	−0.004
07PB1887	171.17	3.423	1.788	1.92	2.17	50	230	1.576	−0.003
11PB6931	157.53	3.151	1.625	1.94	2.28	50	230	1.384	0
11PB6931	156.69	3.134	1.632	1.92	2.2	50	230	1.425	0.019
11PB6931	154.11	3.082	1.641	1.88	2.25	50	230	1.368	0.016
07PB2880	200.73	4.015	2.11	1.9	2.14	50	230	1.877	0.007
07PB2880	198.35	3.967	2.036	1.95	2.19	50	230	1.811	0.004
07PB2880	202.41	4.048	2.119	1.91	2.18	50	230	1.858	0.013
10PB6041	171.75	3.435	1.808	1.9	2.11	50	230	1.629	0.009
10PB6041	190.53	3.811	2.015	1.89	2.15	50	230	1.774	0.004
10PB6041	174.07	3.481	1.826	1.91	2.1	50	230	1.661	−0.008
09PB0698	174.57	3.491	1.825	1.91	2.21	50	230	1.582	−0.006
09PB0698	139.6	2.792	1.451	1.92	2.23	50	230	1.252	−0.018
09PB0698	156.91	3.138	1.623	1.93	2.24	50	230	1.4	0.025
07PB6512	175.31	3.506	1.818	1.93	2.11	50	230	1.661	0.001
07PB6512	192.66	3.853	2.02	1.91	2.13	50	230	1.812	−0.015
07PB6512	174.94	3.499	1.837	1.9	2.12	50	230	1.654	−0.008
12PB8783	156.29	3.126	1.611	1.94	1.68	50	230	1.856	−0.013
12PB8783	153.44	3.069	1.593	1.93	1.69	50	230	1.82	0.004
12PB8783	153.83	3.077	1.588	1.94	1.67	50	230	1.844	0.002
13PB11267	241.17	4.823	2.508	1.92	2.21	50	230	2.18	−0.014
13PB11267	247.17	4.943	2.593	1.91	2.21	50	230	2.242	−0.006
13PB11267	240.73	4.815	2.502	1.92	2.2	50	230	2.186	−0.01
11PB0842	188.39	3.768	1.949	1.93	2.18	50	230	1.729	0.011
11PB0842	179.38	3.588	1.864	1.92	2.13	50	230	1.686	0.006
11PB0842	192	3.84	1.999	1.92	2.16	50	230	1.775	0.006
12PB7846	157.68	3.154	1.636	1.93	2.06	50	230	1.532	−0.038
12PB7846	168.2	3.364	1.765	1.91	2.09	50	230	1.613	−0.03
12PB7846	154.06	3.081	1.593	1.93	2.07	50	230	1.488	−0.032
08PB1543	84.01	1.68	0.863	1.95	2	50	230	0.839	−0.001
08PB1543	84.08	1.682	0.903	1.86	1.93	50	230	0.873	0.006
08PB1543	83.55	1.671	0.859	1.94	1.97	50	230	0.849	0.022
08PB2670	104.94	2.099	1.093	1.92	1.72	50	230	1.219	−0.027
08PB2670	101.87	2.037	1.047	1.95	1.79	50	230	1.14	−0.015
08PB2670	108.54	2.171	1.146	1.89	1.74	50	230	1.245	0.361

**Online-only Table 3:** Quality control of signals from the Illumina Infinium HumanMethylation450 BeadChips.

Index	Sample ID	Sentrix Barcode	Sample Section	Detected CpG (0.01)	DetectRate (0.01)	Loci NOT detected (0.01)	Detected CpG (0.05)	Signal Average GRN	Signal Average RED	Signal P05 GRN	Signal P05 RED	Signal P25 GRN	Signal P25 RED	Signal P50 GRN	Signal P50 RED	Signal P75 GRN	Signal P75 RED	Signal P95 GRN	Signal P95 RED	DNA concentration (ng/µl)	DNA volume (µl)	DNA quantity loaded (ng)
1	09PB8587	9422491005	R01C01	485085	**99.90%**	**492**	485165	3651	8821	211	411	468	1308	1234	4781	6569	15293	11915	26452	29.1	34.3	1000
2	10PB0115	9422491005	R01C02	485131	**99.91%**	**446**	485203	3729	8353	239	476	504	1355	1371	4890	6632	14167	12040	24642	35.1	28.5	1000
3	11PB5152	9422491005	R02C01	485445	**99.97%**	**132**	485494	3957	8787	248	487	544	1407	1495	4956	6994	14999	12769	26193	31.7	31.6	1000
4	10PB2807	9422491005	R02C02	485141	**99.91%**	**436**	485213	4030	8682	265	519	551	1414	1418	4976	7249	14806	13005	25570	39.3	25.4	1000
5	09PB7427	9422491005	R03C01	485435	**99.97%**	**142**	485480	4209	9271	265	524	578	1462	1549	5041	7582	15980	13405	27712	37.6	26.6	1000
6	09PB0906	9422491005	R03C02	485418	**99.97%**	**159**	485480	4418	8669	263	504	567	1331	1551	4307	8127	15071	13857	26491	38	26.3	1000
7	08PB4647	9422491005	R04C01	485267	**99.94%**	**310**	485347	4502	9217	285	579	610	1433	1608	4310	8235	16153	14142	28523	41.7	24	1000
8	09PB0626	9422491005	R04C02	485414	**99.97%**	**163**	485473	4655	9046	294	604	626	1461	1820	4346	8487	15754	14258	27851	32.2	31	1000
9	08PB0411	9422491005	R05C01	485084	**99.90%**	**493**	485181	4437	9338	275	549	605	1459	1661	4635	8053	16226	13947	28595	39.6	25.3	1000
10	10PB0381	9422491005	R05C02	485102	**99.90%**	**475**	485175	4362	9486	288	569	610	1597	1620	5574	7800	16175	13948	27647	46.4	21.6	1000
11	09PB6227	9422491005	R06C01	485382	**99.96%**	**195**	485425	4410	9549	281	553	589	1482	1604	4927	7906	16364	14151	29340	42	23.8	1000
12	09PB1646	9422491005	R06C02	485366	**99.96%**	**211**	485428	4407	9354	289	562	594	1427	1541	4707	7874	16145	14491	28790	53.1	18.8	1000
13	10PB6173	9422491007	R01C01	485081	**99.90%**	**496**	485198	4169	9048	276	577	564	1525	1434	5155	7476	15427	13526	26653	37.2	26.9	1000
14	09PB7511	9422491007	R01C02	485401	**99.96%**	**176**	485457	4103	8717	270	600	549	1400	1516	4274	7427	15087	12850	26602	38.4	26	1000
15	08PB4270	9422491007	R02C01	485133	**99.91%**	**444**	485222	4416	8742	254	523	553	1277	1545	3909	8126	15388	13836	27270	35.8	27.9	1000
16	10PB2738	9422491007	R02C02	485447	**99.97%**	**130**	485484	4170	8703	244	500	524	1274	1418	4146	7634	15196	13255	26902	39.8	25.1	1000
17	09PB6550	9422491007	R03C01	485121	**99.91%**	**456**	485190	4487	9178	252	491	567	1402	1645	4942	8185	15855	14144	27522	37.2	26.9	1000
18	11PB13477	9422491007	R03C02	485445	**99.97%**	**132**	485482	4493	9192	254	489	563	1372	1585	4874	8242	15934	14150	27585	40.4	24.8	1000
19	11PB5972	9422491007	R04C01	485271	**99.94%**	**306**	485370	4624	9269	245	460	571	1359	1611	4755	8513	16163	14666	28159	40	25	1000
20	12PB0638	9422491007	R04C02	485429	**99.97%**	**148**	485475	4686	9088	248	465	577	1313	1808	4322	8591	15933	14474	28215	36.4	27.4	1000
21	09PB2752	9422491007	R05C01	485385	**99.96%**	**192**	485447	4260	9676	245	452	545	1471	1365	5767	7630	16552	14303	28418	45.4	22	1000
22	10PB5506	9422491007	R05C02	485065	**99.89%**	**512**	485147	4459	9472	259	478	568	1441	1486	5264	8187	16322	14343	28140	40.9	24.4	1000
23	10PB2885	9422491007	R06C01	485093	**99.90%**	**484**	485164	4520	9316	254	498	560	1371	1642	4558	8242	16026	14329	29148	41.3	24.2	1000
24	12PB8783	9422491007	R06C02	485129	**99.91%**	**448**	485196	4712	9357	269	514	592	1406	1796	4578	8646	16181	14538	29097	41	24.4	1000
25	09PB0858	9422491020	R01C01	485018	**99.88%**	**559**	485139	4272	8893	253	492	569	1451	1563	4823	7669	15287	13647	26617	25.8	37	953
26	09PB6581	9422491020	R01C02	485384	**99.96%**	**193**	485475	4184	8923	286	608	591	1511	1627	4542	7545	15345	12978	27054	36.6	27.4	1000
27	09PB4164	9422491020	R02C01	485391	**99.96%**	**186**	485452	4513	9004	282	548	614	1455	1640	4721	8206	15598	14233	27006	38.5	26	1000
28	11PB1528	9422491020	R02C02	485392	**99.96%**	**185**	485441	4659	9155	325	664	679	1684	1939	5263	8352	15416	14308	26900	30.7	32.6	1000
29	10PB0279	9422491020	R03C01	485433	**99.97%**	**144**	485494	4537	9291	278	519	614	1470	1639	4958	8257	16096	14340	27738	35.1	28.5	1000
30	11PB2066	9422491020	R03C02	485413	**99.97%**	**164**	485448	4706	9292	309	616	647	1518	1716	4654	8620	16096	14600	28190	40.8	24.5	1000
31	08PB8276	9422491020	R04C01	485409	**99.97%**	**168**	485471	4648	9277	275	507	618	1445	1748	4706	8502	16177	14433	28127	34.6	28.9	1000
32	09PB0700	9422491020	R04C02	485424	**99.97%**	**153**	485490	4720	9423	297	580	644	1473	1719	4545	8669	16485	14694	28790	35.5	28.2	1000
33	10PB3262	9422491020	R05C01	485444	**99.97%**	**133**	485488	4615	9232	289	547	638	1492	1745	4801	8327	15984	14533	27880	31.2	32.1	1000
34	10PB10381	9422491020	R05C02	485446	**99.97%**	**131**	485488	5023	9383	293	585	684	1620	2617	4853	9064	15967	14648	28812	37.5	26.7	1000
35	10PB0918	9422491020	R06C01	485257	**99.93%**	**320**	485348	4475	10116	276	605	611	1577	1647	4880	8149	17638	14105	31126	41.2	24.3	1000
36	08PB3739	9422491020	R06C02	485239	**99.93%**	**338**	485319	4613	10049	301	647	627	1595	1617	4957	8447	17388	14584	30731	31.5	31.7	1000
37	08PB8771	9422491034	R01C01	485386	**99.96%**	**191**	485453	3758	7873	255	509	528	1333	1372	4074	6866	13575	11652	23793	36	27.8	1000
38	08PB0722	9422491034	R01C02	485361	**99.96%**	**216**	485437	3801	7933	269	543	541	1357	1417	4319	6913	13526	11730	23654	34.8	28.8	1000
39	10PB3589	9422491034	R02C01	485209	**99.92%**	**368**	485286	4115	8595	304	606	611	1487	1550	4665	7498	14742	12619	25440	39.8	25.1	1000
40	11PB5602	9422491034	R02C02	484874	**99.86%**	**703**	485029	4161	8577	316	643	638	1522	1758	4559	7531	14611	12501	25709	29.6	33.8	1000
41	10PB1144	9422491034	R03C01	485424	**99.97%**	**153**	485472	4185	8568	292	604	604	1493	1646	4622	7619	14666	12746	25441	40.2	24.9	1000
42	07PB2880	9422491034	R03C02	485306	**99.94%**	**271**	485380	4248	8646	298	609	612	1425	1585	4136	7802	15084	12997	26260	36	27.8	1000
43	08PB6113	9422491034	R04C01	485165	**99.92%**	**412**	485224	4396	8633	282	597	636	1532	2331	4566	7855	14713	12801	26047	41.2	24.3	1000
44	08PB8031	9422491034	R04C02	485024	**99.89%**	**553**	485111	4244	9164	304	628	623	1563	1559	4902	7748	15745	13186	27171	40	25	1000
45	08PB5124	9422491034	R05C01	485023	**99.89%**	**554**	485113	4302	9383	314	673	643	1682	1621	5324	7816	16015	13332	27359	38.3	26.1	1000
46	10PB8858	9422491034	R05C02	484942	**99.87%**	**635**	485047	4480	9434	312	641	651	1589	1715	4925	8146	16221	13748	28290	25.7	37	952
47	11PB6931	9422491034	R06C01	485002	**99.88%**	**575**	485084	4388	8936	295	611	625	1507	1749	4663	7931	15268	13493	27067	39.3	25.5	1000
48	07PB1887	9422491034	R06C02	485257	**99.93%**	**320**	485386	4430	8773	312	644	624	1457	1597	4110	8133	15201	13742	27127	28.6	34.9	1000
49	09PB7242	9422491041	R01C01	485475	**99.98%**	**102**	485515	4030	8303	253	505	541	1328	1472	4189	7230	14294	12917	25500	49.4	20.2	1000
50	08PB1082	9422491041	R01C02	485440	**99.97%**	**137**	485500	3896	8119	243	499	516	1203	1404	3523	6986	14179	12587	26004	65.5	15.3	1000
51	13PB11267	9422491041	R02C01	485461	**99.98%**	**116**	485508	4358	8579	286	591	601	1309	1674	3600	7836	14966	13797	27693	96.2	10.4	1000
52	10PB0488	9422491041	R02C02	485393	**99.96%**	**184**	485440	4063	8812	278	607	572	1390	1506	4066	7269	15170	12999	27791	79.5	12.6	1000
53	08PB1543	9422491041	R03C01	485184	**99.92%**	**393**	485277	4430	9034	289	568	606	1466	1603	4817	7994	15546	14115	27126	43.2	23.1	1000
54	10PB1329	9422491041	R03C02	485435	**99.97%**	**142**	485494	4350	9460	297	654	628	1635	1822	5190	7772	16154	13517	28100	42.5	23.5	1000
55	09PB2750	9422491041	R04C01	485507	**99.99%**	**70**	485529	4335	8977	265	503	579	1381	1569	4791	7802	15540	13885	27005	66.3	15.1	1000
56	08PB2670	9422491041	R04C02	485179	**99.92%**	**398**	485260	4422	8870	272	549	588	1326	1714	3944	8009	15522	13844	28010	71.5	14	1000
57	10PB2975	9422491041	R05C01	485372	**99.96%**	**205**	485425	4494	9474	307	631	625	1523	1591	4834	8146	16368	14238	28682	44.2	22.6	1000
58	10PB8700	9422491041	R05C02	485307	**99.94%**	**270**	485386	4312	9582	300	636	603	1563	1487	5059	7783	16472	13897	28798	43.1	23.2	1000
59	11PB11934	9422491041	R06C01	485104	**99.90%**	**473**	485200	4608	9536	299	610	642	1561	1874	4846	8241	16270	14388	29437	64.4	15.5	1000
60	09PB1757	9422491041	R06C02	485400	**99.96%**	**177**	485459	4431	9729	306	659	624	1553	1613	4741	8004	16875	14067	29859	53.4	18.7	1000
61	09PB5837	9422491055	R01C01	485459	**99.98%**	**118**	485502	3217	7693	204	424	445	1208	1194	3928	5629	13163	10600	23798	88.3	11.3	1000
62	08PB3517	9422491055	R01C02	485439	**99.97%**	**138**	485489	3929	8063	258	525	533	1302	1388	3914	7164	14029	12406	24669	46.1	21.7	1000
63	11PB1958	9422491055	R02C01	485462	**99.98%**	**115**	485507	4087	8327	270	518	571	1360	1529	4477	7245	14202	13178	25164	58.5	17.1	1000
64	11PB0842	9422491055	R02C02	485106	**99.90%**	**471**	485194	4338	8399	277	551	589	1367	1715	4122	7854	14462	13482	25922	56.5	17.7	1000
65	10PB5849	9422491055	R03C01	485223	**99.93%**	**354**	485289	4128	8836	260	475	558	1395	1404	5096	7368	15149	13647	25970	51.9	19.3	1000
66	10PB6041	9422491055	R03C02	485437	**99.97%**	**140**	485494	4513	8485	265	509	581	1273	1584	3899	8305	14945	14174	26289	45	22.2	1000
67	08PB5904	9422491055	R04C01	485423	**99.97%**	**154**	485482	4330	8915	275	544	594	1372	1587	4219	7778	15481	13952	27851	89.1	11.2	1000
68	10PB6266	9422491055	R04C02	485433	**99.97%**	**144**	485477	4590	8741	274	523	590	1309	1560	4033	8490	15369	14462	27039	45.3	22.1	1000
69	08PB7544	9422491055	R05C01	485395	**99.96%**	**182**	485454	4255	8991	278	580	602	1429	1638	4295	7594	15540	13576	28130	64.5	15.5	1000
70	07PB6512	9422491055	R05C02	485423	**99.97%**	**154**	485487	4583	9045	278	553	614	1441	1680	4550	8299	15701	14566	27590	77.3	12.9	1000
71	09PB0698	9422491055	R06C01	485074	**99.90%**	**503**	485153	4220	9005	276	579	580	1397	1635	4270	7567	15469	13418	28391	76	13.2	1000
72	12PB7846	9422491055	R06C02	485416	**99.97%**	**161**	485470	4699	8945	288	575	622	1402	1741	4189	8546	15426	14862	28138	23.3	37	863
