# Supplementary material for: DNA methylation of chronic lymphocytic leukemia with differential response to chemotherapy
Source: Sci Data. 2020 May 1;7:133. doi: 10.1038/s41597-020-0456-0 (PMC7195470; doi:10.1038/s41597-020-0456-0)
Supplement: Supplementary file 1 [file 41597_2020_456_MOESM1_ESM.pdf]

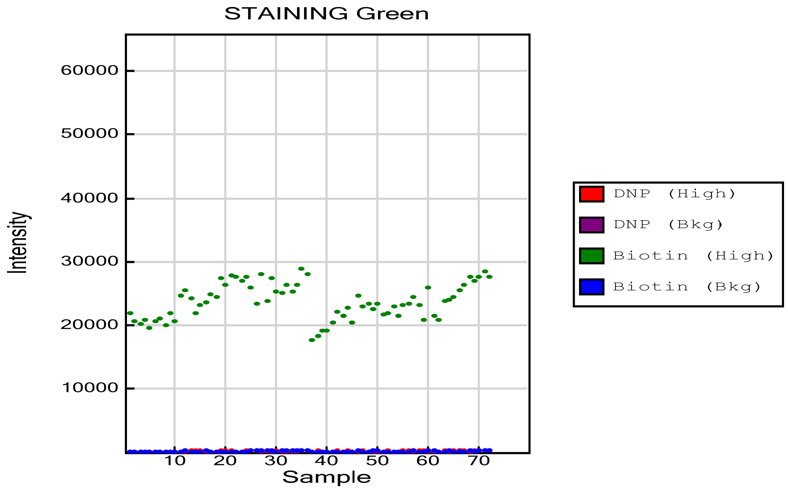
STAINING Green
Intensity
60000
50000
40000
30000
20000
10000
10
20
30
40
50
60
70
Sample
DNP (High)
DNP (Bkg)
Biotin (High)
Biotin (Bkg)

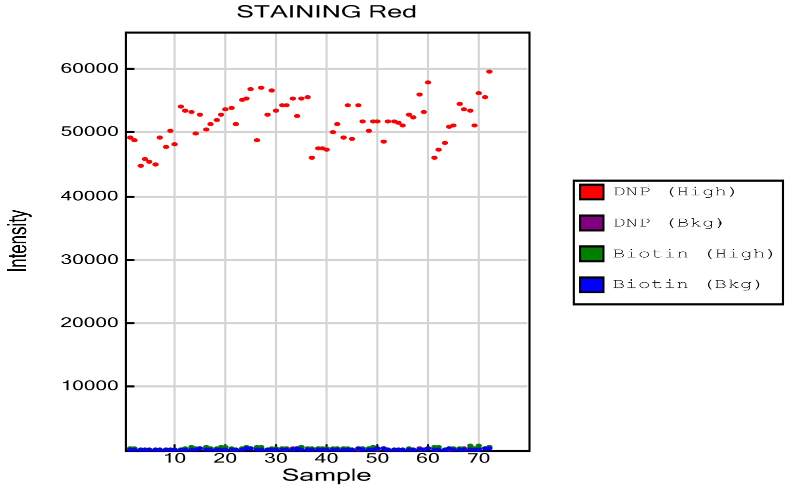
STAINING Red
Intensity
60000
50000
40000
30000
20000
10000
10
20
30
40
50
60
70
Sample
DNP (High)
DNP (Bkg)
Biotin (High)
Biotin (Bkg)

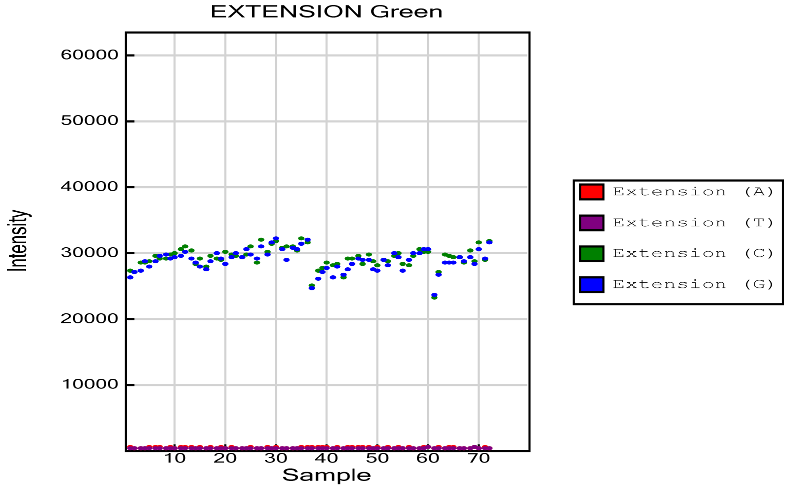
EXTENSION Green
Intensity
60000
50000
40000
30000
20000
10000
10
20
30
40
50
60
70
Sample
Extension (A)
Extension (T)
Extension (C)
Extension (G)

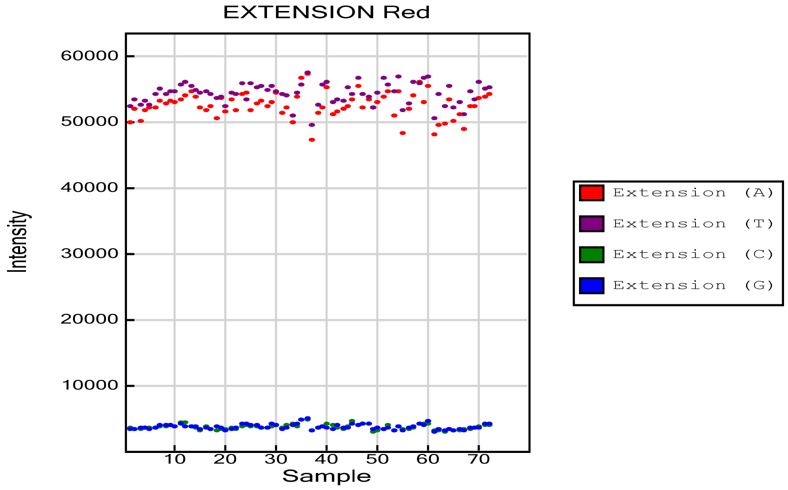
EXTENSION Red
Intensity
60000
50000
40000
30000
20000
10000
10
20
30
40
50
60
70
Sample
Extension (A)
Extension (T)
Extension (C)
Extension (G)

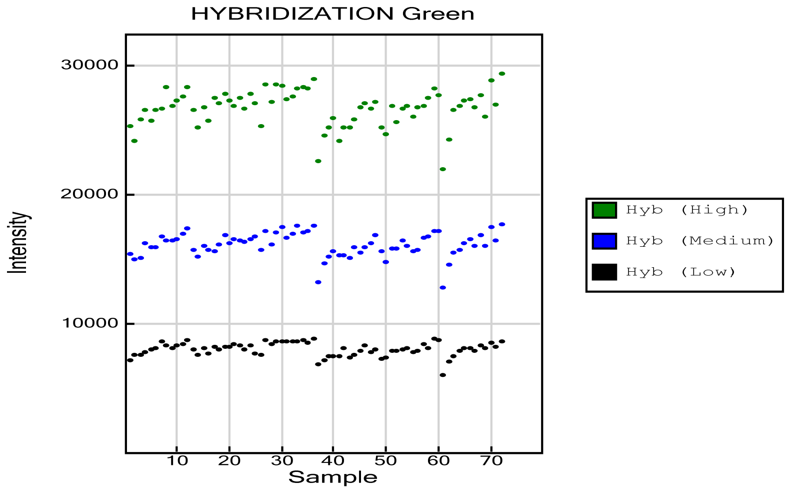
HYBRIDIZATION Green
Intensity
30000
20000
10000
10
20
30
40
50
60
70
Sample
Hyb (High)
Hyb (Medium)
Hyb (Low)

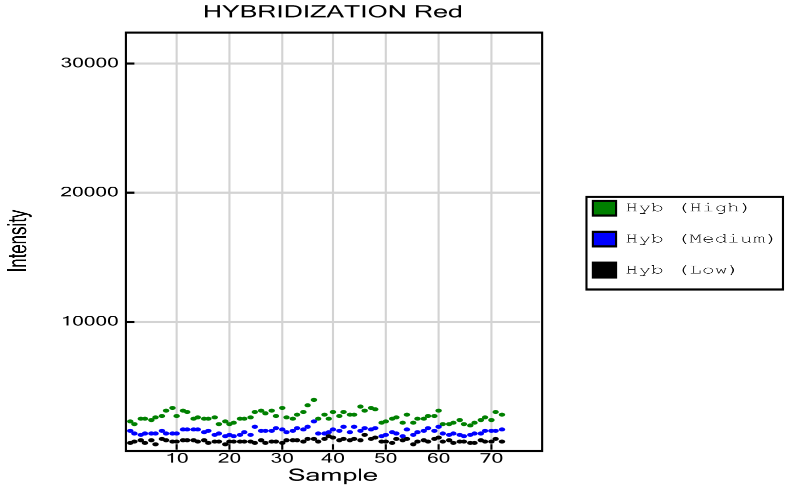
HYBRIDIZATION Red
Intensity
30000
20000
10000
10
20
30
40
50
60
70
Sample
Hyb (High)
Hyb (Medium)
Hyb (Low)

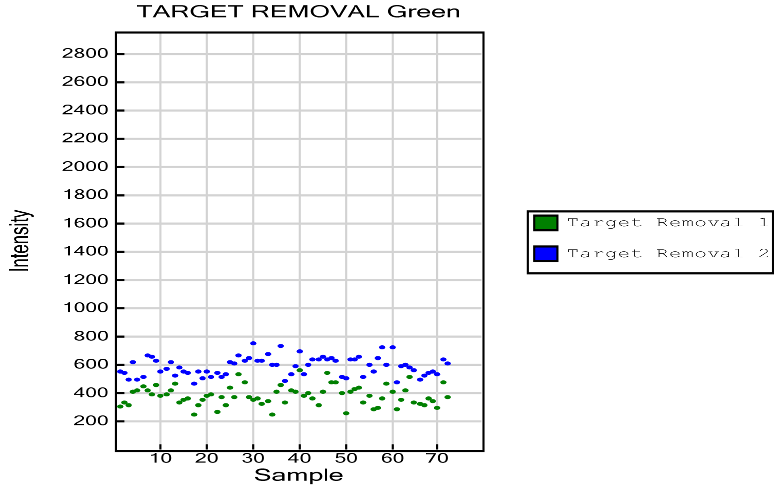
TARGET REMOVAL Green
Intensity
2800
2600
2400
2200
2000
1800
1600
1400
1200
1000
800
600
400
200
10
20
30
40
50
60
70
Sample
Target Removal 1
Target Removal 2

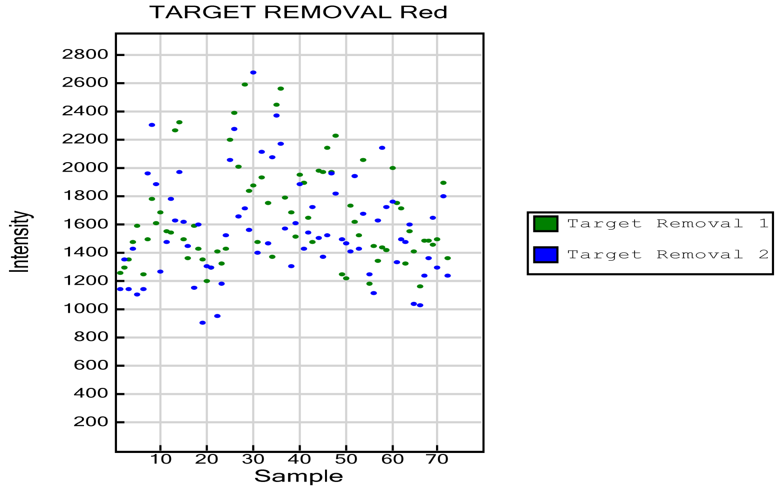
TARGET REMOVAL Red
Intensity
2800
2600
2400
2200
2000
1800
1600
1400
1200
1000
800
600
400
200
10
20
30
40
50
60
70
Sample
Target Removal 1
Target Removal 2

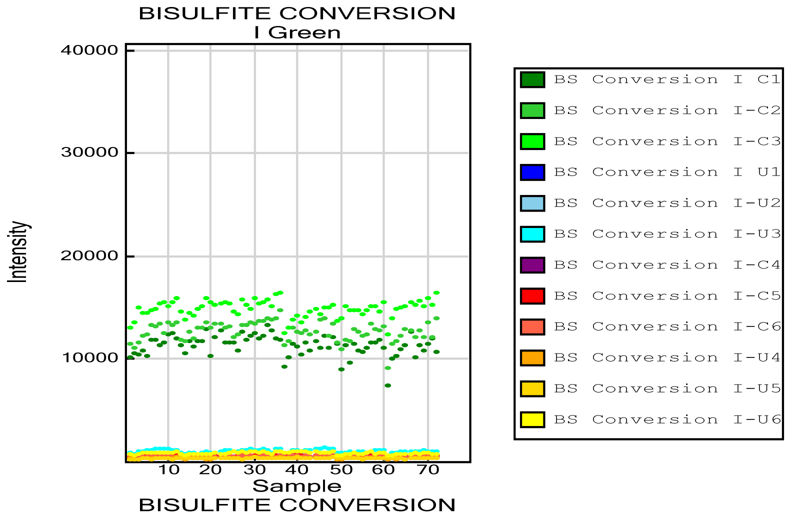
BISULFITE CONVERSION
I Green
Intensity
40000
30000
20000
10000
10 20 30 40 50 60 70
Sample
BS Conversion I C1
BS Conversion I-C2
BS Conversion I-C3
BS Conversion I U1
BS Conversion I-U2
BS Conversion I-U3
BS Conversion I-C4
BS Conversion I-C5
BS Conversion I-C6
BS Conversion I-U4
BS Conversion I-U5
BS Conversion I-U6

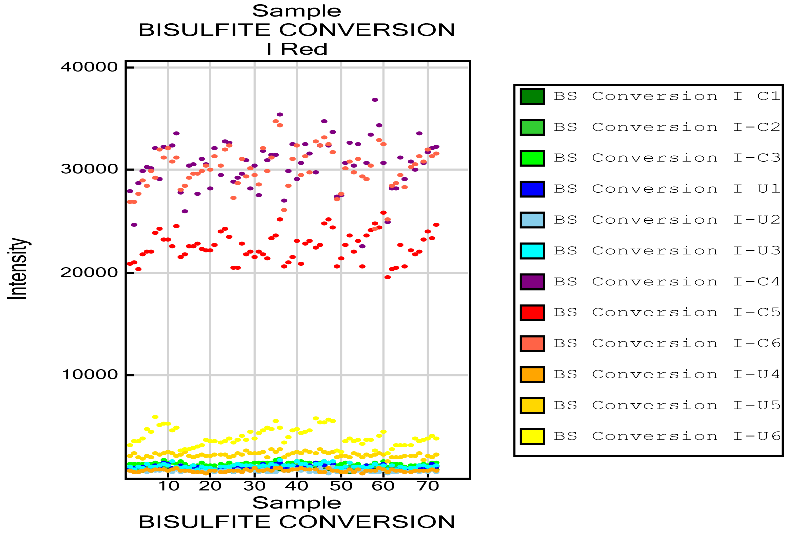
BISULFITE CONVERSION
I Red
Intensity
40000
30000
20000
10000
10 20 30 40 50 60 70
Sample
BS Conversion I C1
BS Conversion I-C2
BS Conversion I-C3
BS Conversion I U1
BS Conversion I-U2
BS Conversion I-U3
BS Conversion I-C4
BS Conversion I-C5
BS Conversion I-C6
BS Conversion I-U4
BS Conversion I-U5
BS Conversion I-U6

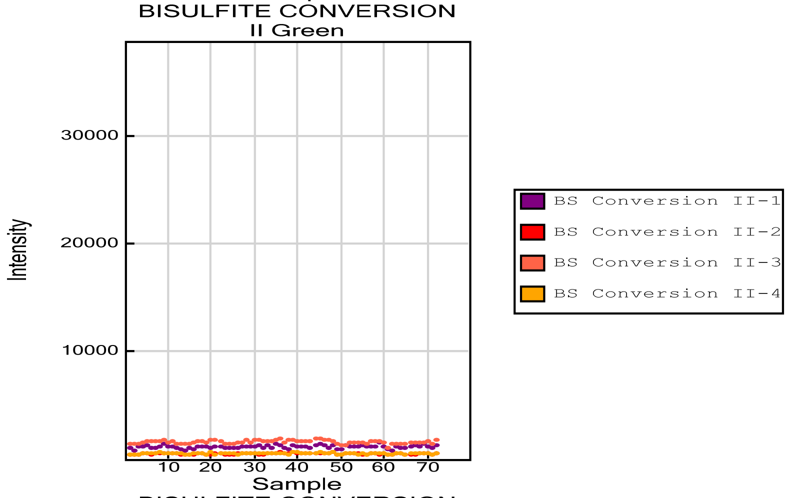
BISULFITE CONVERSION
II Green
Intensity
30000
20000
10000
10 20 30 40 50 60 70
Sample
BS Conversion II-1
BS Conversion II-2
BS Conversion II-3
BS Conversion II-4

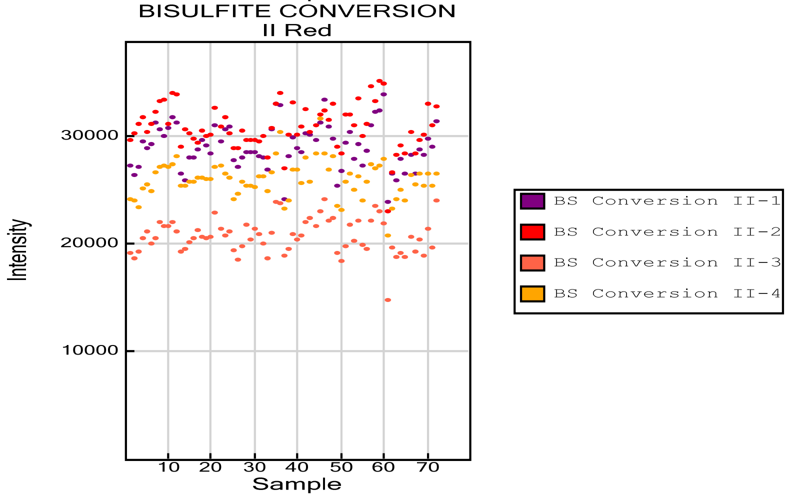
BISULFITE CONVERSION
II Red
Intensity
30000
20000
10000
10 20 30 40 50 60 70
Sample
BS Conversion II-1
BS Conversion II-2
BS Conversion II-3
BS Conversion II-4

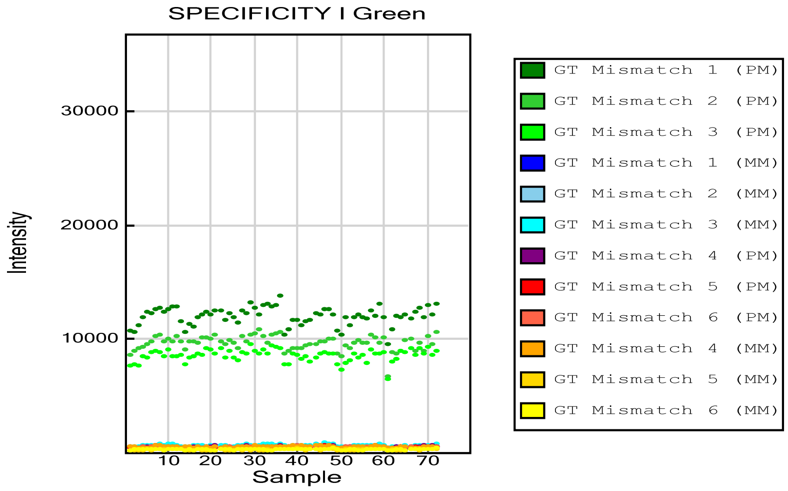
SPECIFICITY I Green
Intensity
30000
20000
10000
10
20
30
40
50
60
70
Sample
GT Mismatch 1 (PM)
GT Mismatch 2 (PM)
GT Mismatch 3 (PM)
GT Mismatch 1 (MM)
GT Mismatch 2 (MM)
GT Mismatch 3 (MM)
GT Mismatch 4 (PM)
GT Mismatch 5 (PM)
GT Mismatch 6 (PM)
GT Mismatch 4 (MM)
GT Mismatch 5 (MM)
GT Mismatch 6 (MM)

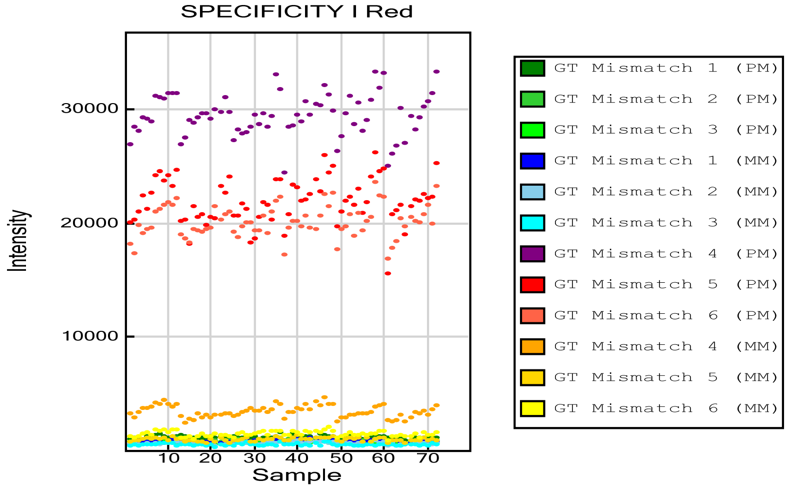
SPECIFICITY I Red
Intensity
30000
20000
10000
10
20
30
40
50
60
70
Sample
GT Mismatch 1 (PM)
GT Mismatch 2 (PM)
GT Mismatch 3 (PM)
GT Mismatch 1 (MM)
GT Mismatch 2 (MM)
GT Mismatch 3 (MM)
GT Mismatch 4 (PM)
GT Mismatch 5 (PM)
GT Mismatch 6 (PM)
GT Mismatch 4 (MM)
GT Mismatch 5 (MM)
GT Mismatch 6 (MM)

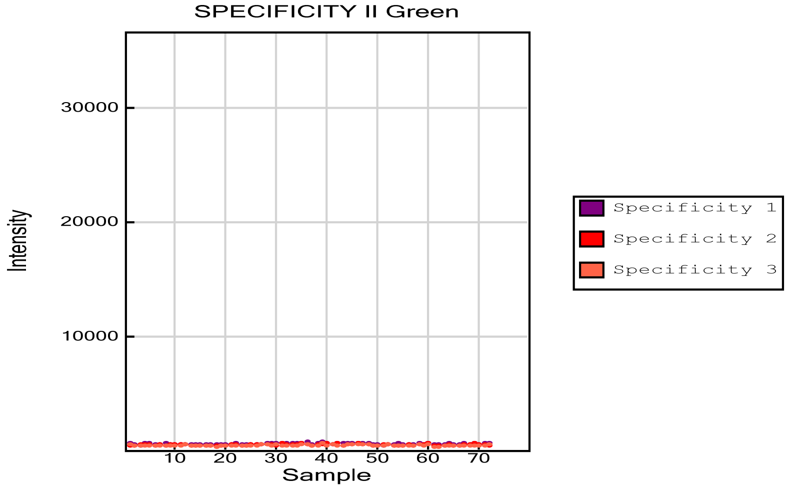
SPECIFICITY II Green
Intensity
30000
20000
10000
10
20
30
40
50
60
70
Sample
Specificity 1
Specificity 2
Specificity 3

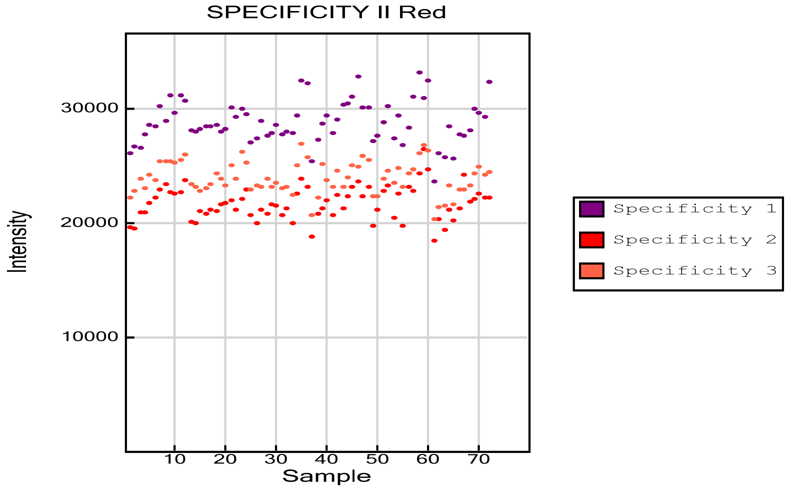
SPECIFICITY II Red
Intensity
30000
20000
10000
10
20
30
40
50
60
70
Sample
Specificity 1
Specificity 2
Specificity 3

NON-POLYMORPHIC Green

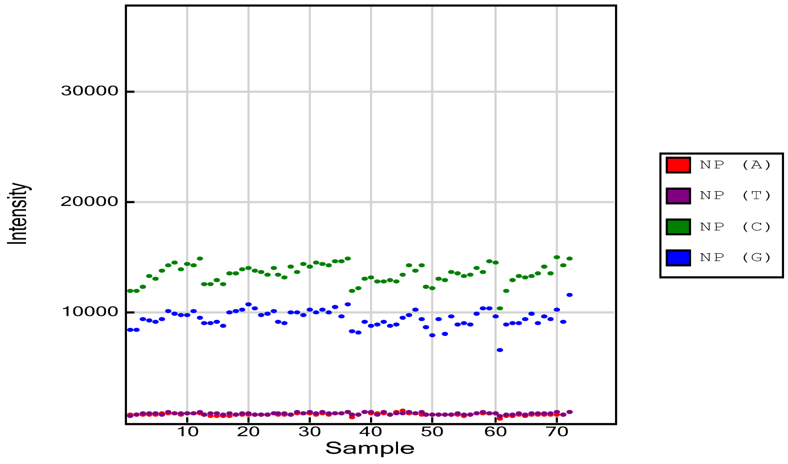

NON-POLYMORPHIC Red

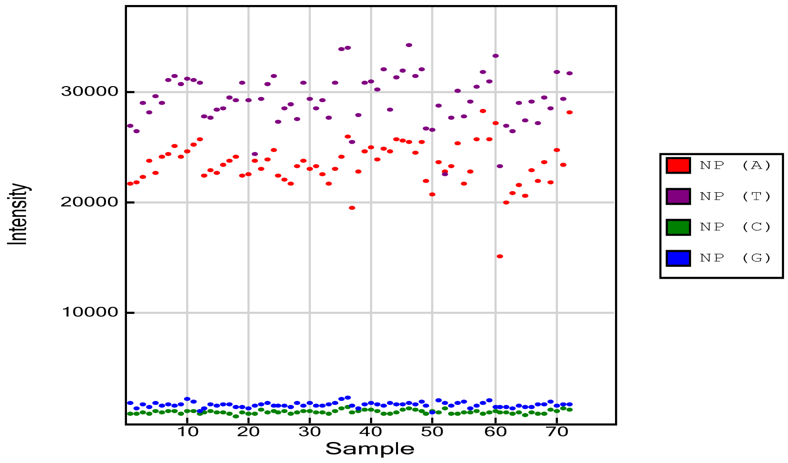

NEGATIVE Green

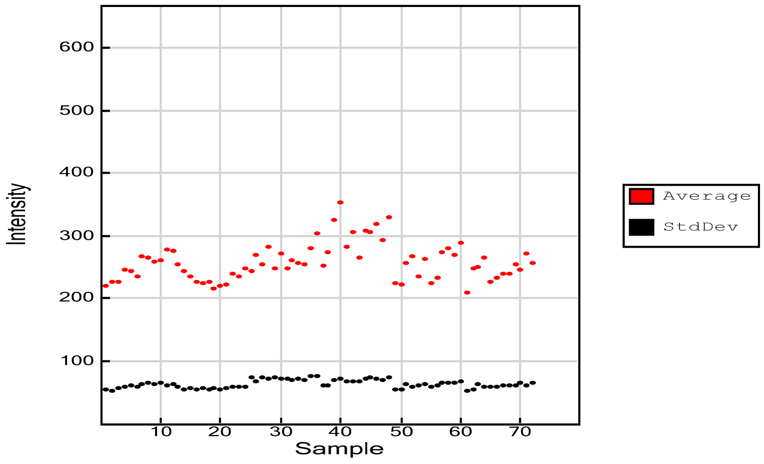

NEGATIVE Red

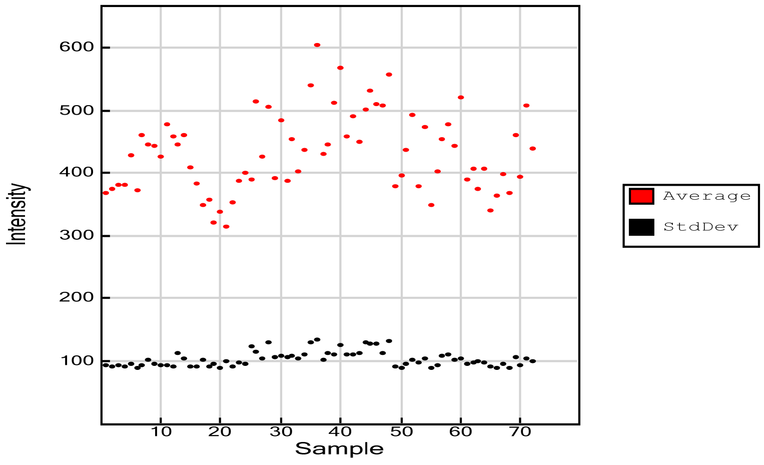

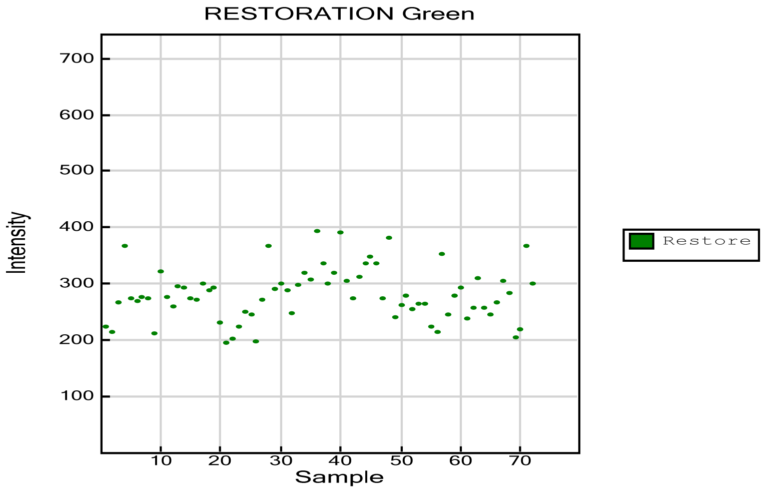
RESTORATION Green
Intensity
Sample
Restore

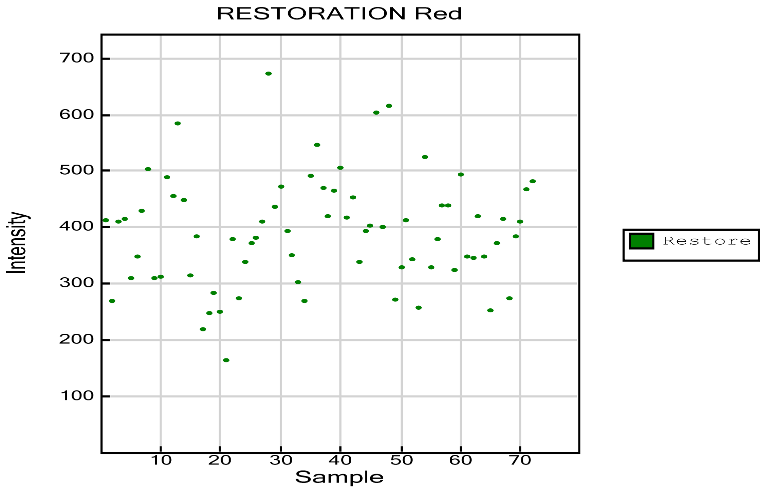
RESTORATION Red
Intensity
Sample
Restore
